# Hybrid Approach for Predicting Coreceptor Used by HIV-1 from Its V3 Loop Amino Acid Sequence

**DOI:** 10.1371/journal.pone.0061437

**Published:** 2013-04-15

**Authors:** Ravi Kumar, Gajendra P. S. Raghava

**Affiliations:** Bioinformatics Centre, Institute of Microbial Technology (Council of Scientific and Industrial Research), Chandigarh, India; New York University, United States of America

## Abstract

**Background:**

HIV-1 infects the host cell by interacting with the primary receptor CD4 and a coreceptor CCR5 or CXCR4. Maraviroc, a CCR5 antagonist binds to CCR5 receptor. Thus, it is important to identify the coreceptor used by the HIV strains dominating in the patient. In past, a number of experimental assays and *in-silico* techniques have been developed for predicting the coreceptor tropism. The prediction accuracy of these methods is excellent when predicting CCR5(R5) tropic sequences but is relatively poor for CXCR4(X4) tropic sequences. Therefore, any new method for accurate determination of coreceptor usage would be of paramount importance to the successful management of HIV-infected individuals.

**Results:**

The dataset used in this study comprised 1799 R5-tropic and 598 X4-tropic third variable (V3) sequences of HIV-1. We compared the amino acid composition of both types of V3 sequences and observed that certain types of residues, e.g., Asparagine and Isoleucine, were preferred in R5-tropic sequences whereas residues like Lysine, Arginine, and Tryptophan were preferred in X4-tropic sequences. Initially, Support Vector Machine-based models were developed using amino acid composition, dipeptide composition, and split amino acid composition, which achieved accuracy up to 90%. We used BLAST to discriminate R5- and X4-tropic sequences and correctly predicted 93.16% of R5- and 75.75% of X4-tropic sequences. In order to improve the prediction accuracy, a Hybrid model was developed that achieved 91.66% sensitivity, 81.77% specificity, 89.19% accuracy and 0.72 Matthews Correlation Coefficient. The performance of our models was also evaluated on an independent dataset (256 R5- and 81 X4-tropic sequences) and achieved maximum accuracy of 84.87% with Matthews Correlation Coefficient 0.63.

**Conclusion:**

This study describes a highly efficient method for predicting HIV-1 coreceptor usage from V3 sequences. In order to provide a service to the scientific community, a webserver HIVcoPred was developed (http://www.imtech.res.in/raghava/hivcopred/) for predicting the coreceptor usage.

## Introduction

Human Immunodeficiency Virus (HIV) is a retrovirus which infects the human immune cells - mainly CD4+ Helper T lymphocytes, monocytes, macrophages and dendritic cells. When left untreated, the HIV infected subjects may eventually develop Acquired Immunodeficiency Syndrome (AIDS). There are two types of HIV strains, HIV-1 and HIV-2, the type-1 (Group M) is responsible for the pandemic form and has been reported in every country of the world, whereas HIV-2 is mainly restricted to West Africa [1].

The infection of human cells by HIV is initiated by the molecular interactions between the surface receptors of the host and the pathogen. The core interactions are conserved for all the HIV infections and mediated through the HIV surface protein gp120 (glycoprotein 120). This glycoprotein interacts with the CD4 receptor present on the surface of immune cells thereby initiating the mechanistic pathway leading to the infection by HIV. The interaction with CD4 receptor induces immediate conformational changes in gp120 protein that leads to the complete exposure of the third variable (V3) loop. The exposed loop further interacts with either of the two coreceptors present on human cells, *i.e*. CCR5 or CXCR4 [2], [3]. This interaction is required for the successful fusion of cell membrane of HIV pathogen and host T_H_ cells, which ultimately results in the transmission of the viral genetic material into the host cells [4].

The coreceptor tropism is defined as the ability of a particular HIV-1 virus to infect a target cell using a specific coreceptor. The HIV-1 strains which use CCR5 as coreceptor are termed as R5-tropic, whereas the strains that utilize CXCR4 as coreceptor are called X4-tropic viruses. R5X4 or dual tropic strains constitute a third major class as they can use either of these two coreceptors [5]. The difference in the coreceptor usage points towards the physiological differences in the pathogenicity, tissue tropism and transmissibility of the virus *in-vivo* [6]. It has been reported that in majority of the infected subjects, the HIV pathogen primarily used the coreceptor CCR5 in order to initiate the infection pathway [7], [8]. During course of infection, the coreceptor usage preference of HIV changes from CCR5 to CXCR4 in approx. 50% of the infected individuals. This switchover from R5- to X4-tropism was found to be associated with the accelerated CD4+ T-cell decline and the rapid progression to AIDS [9], [10].

Maraviroc (Selzentry/Celsentri) is a FDA approved drug that targets the CCR5 coreceptor. Binding of the drug to CCR5 leads to the conformational changes in the extracellular loops of CCR5, making them inaccessible to the V3 loop of gp120 protein [11], [12]. Since there may be a heterogeneous population of HIV-1 in an infected person, it is essential to determine whether the subject is exclusively harbouring R5-tropic strains before the use of CCR5 antagonist *e.g.* Maraviroc [13]. Primarily, there are two types of methods to determine the HIV-1 tropism – (1) Recombinant Phenotypic Assays (RPA) *e.g*. Monogram Trofile Tropism Assay (2) Genotypic methods (*in-silico* approaches). In the RPA method, pseudo viruses or infectious recombinant viruses generated from the patient’s plasma, having either full or partial-length viral envelope regions and tested on the indicator cell lines [14]. These cell lines express CD4 and either CCR5 or CXCR4 on their cell surfaces. Based upon the coreceptor used by virus to infect cell lines, the coreceptor tropism is determined [15]. Although the recombinant phenotypic assays are able to distinguish between pure R5, R5X4 and pure X4 populations, these are expensive, laborious, time consuming as well as dependent on the sample availability [16], [17].

On the other hand, *in-silico* based genotypic methods require the HIV protein sequences (mainly the V3 loop of the gp120 protein) to predict the coreceptor tropism. A number of studies have reported that coreceptor usage is largely determined by the sequence of V3 loop [18], [19], [20]. It is highly specific as it has been shown that even a single amino acid substitution in the V3 loop may alter the coreceptor usage by HIV-1 [21]. The 11/25 charge rule was the first genotypic method which predicted the CXCR4 coreceptor usage based on the presence of basic (positively charged) amino acids, *e.g*. Lysine or Arginine, at 11^th^ or 25^th^ position of the V3 loop [22], [23], [24]. Successive studies based on the machine learning approaches used various methods such as Neural Networks [25], [26], Support Vector Machine (SVM) [27], Position Specific Scoring Matrix [28], [29], Random Forest [30], Structural Descriptors [31], distant kernel segments [32], Logistic regression [33] and Decision rule based studies [34]. Clinical datasets have been used with sequence information for developing better SVM models [35]. Most of these studies are exclusively based on the V3 region of gp120 protein but the regions other than V3 are also shown to be important in the prediction of coreceptor usage [36], [37], [38]. Among the other regions, V1, V2, C4 regions of gp120 and gp41 protein are known to play an important role in the determination of the coreceptor usage [39], [40]. It has been reported that the switch to CXCR4-phenotype was associated with an increase in the net positive charge in the V1/V2 stem [41]. The loss of N-glycosylation sites has been associated with the X4 tropism [41] [42]. It has been found that, in addition to the V3 loop, the amino acid variation at residue 440 in the C4 region of gp120 protein is clearly linked with the usage of CXCR4 as coreceptor [43]. Though important in the coreceptor tropism, the lack of sufficient data from these regions (*e.g.* V1, V2) has been the main hindrance for model development and prediction of the coreceptor usage [38]. Recently, it has been reported that genotypic prediction of coreceptor usage was improved with the incorporation of V2 loop sequences’ information along with V3 sequences [21].

Although the earlier methods could predict the CCR5 usage with high accuracy (∼ 95%), the accuracy for CXCR4 usage prediction was relatively poor. It is still a challenge to develop a prediction method with high accuracy for CXCR4 usage. In order to predict the coreceptor usage with high accuracy, we analyzed 1799 R5-tropic and 598 X4-tropic V3 sequences (R5X4 included) and consequently, developed various SVM models. We used a number of input features for various model developments and finally developed a Hybrid model consisting of SAAC and BLAST approaches, which predicted the CCR5 and CXCR4 coreceptor usage with high accuracy (approx. 89.19%).

## Results

It is a challenge to discriminate between the V3 sequences from R5- and X4-tropic viruses. We need to represent the V3 sequences by vectors having numerical values in order to discriminate between the two types of V3 sequences. These vectors, representing the distinct features of the V3 sequences, are used to develop the SVM models. In order to develop the best model for discriminating the R5- and X4-tropic sequences, we optimized the SVM parameters. Following is a brief description of the features used for developing the prediction models.

### Amino Acid Composition (AAC) Based SVM Model

**Figure 1:**
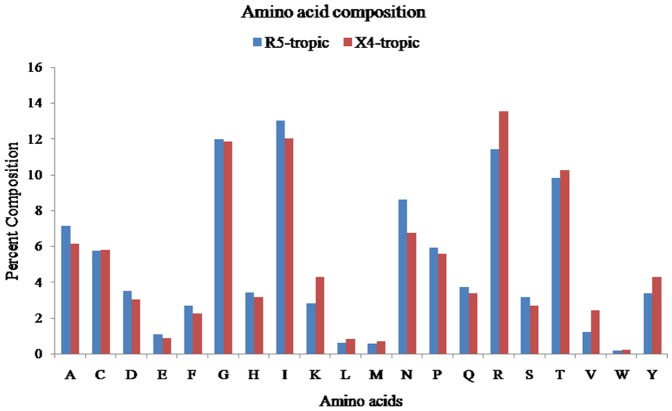
Amino acid composition comparisons of two types of V3 sequence. The Blue bar representing R5-tropic and red bar representing X4-tropic V3 sequences.

**Figure 2:**
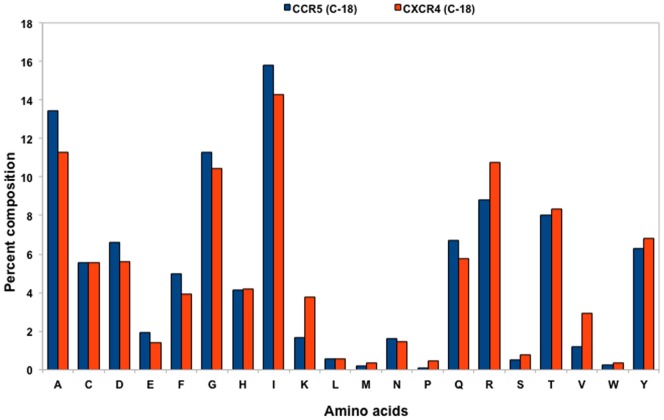
The composition of physico-chemical properties of R5- and X4-tropic V3 sequences. The blue bar representing R5-tropic and red bar representing X4-tropic V3 sequences.

Previously, it has been shown that even a single amino acid mutation in the V3 sequence can alter the coreceptor tropism [21]. Taking this into consideration, the frequency of each of the 20 amino acids was calculated for each R5- and X4-tropic sequences. It was found that certain types of residues are preferred in each tropic class, *e.g*. Lys and Arg are present at higher frequencies in the X4-tropic while the frequency of occurrence of Asn was relatively higher in the R5-tropic sequences. The overall composition of amino acids L (0.82%), M (0.70%), T (10.27%), V (2.41%), W (0.20%) and Y (4.30%) was higher in the X4-tropic whereas A (7.14%), D (3.49%), E (1.10%), F (2.67%), G (11.98%), H (3.43%), I (13.03%), N (8.62%), P (5.94%), Q (3.72%) and S (3.17%) were at higher proportion in the R5-tropic sequences (Figure 1). From the amino acid composition (physico-chemical properties), it is evident that the X4 sequences are primarily dominated by positively charged, large amino acids whereas the R5 sequences show the preference of overall more charged residues (mostly negatively charged) along with small and neutral amino acids (Figure 2). The AAC feature has been previously used to classify different categories of proteins and to develop prediction models [44], [45]. As significant differences in the AAC of R5- and X4-tropic V3 sequences were observed; this deemed possible to use AAC for discriminating the two types of sequences. The SVM-based classifier has been developed using AAC of V3 sequences and achieved 85.82% accuracy with sensitivity of 88.77% and specificity of 76.92% (Table 1, Table S1). Dipeptide composition (DPC) based methods have been shown to be more successful than AAC based methods for the classification of proteins [46]. It is due to the fact that DPC incorporates AAC as well as the local order of amino acids. Thus, a SVM-based classifier was developed using DPC which achieved maximum accuracy of 90.24% with 93.50% sensitivity and 80.43% specificity (Table 1, Table S2). Split Amino Acid Composition (SAAC) has been used successfully in the past to differentiate the proteins that have a signal peptide at the N- or C-terminal [47]. We systematically analyzed the residues at the N- and C-terminal of the R5- and X4-tropic V3 sequences and found significant differences in the AAC of these residues (Figure S1, S2). In order to utilize the compositional difference in the termini of R5- and X4-tropic sequences, we developed SVM modules using SAAC. In case of SAAC, we divided the V3 sequences into two nearly equal parts and calculated the AAC of each part separately. Finally, the input vectors of 40 dimensions have been used to build the SVM models. This approach achieved 88.94% sensitivity, 81.44% specificity, 87.07% accuracy and 0.67 Matthews Correlation Coefficient (MCC) (Table 1, Table S3).

**Table 1:** The performance of various SVM models developed by using Amino Acid, Dipeptide and Split Amino Acid Composition based input vectors.

Method	Threshold	Sensitivity	Specificity	Accuracy	MCC
**AAC**	0.4	88.77	76.92	85.82	0.64
**DPC**	0.2	93.50	80.43	90.24	0.74
**SAAC**	0.4	88.94	81.44	87.07	0.67

### Basic Local Alignment Search Tool (BLAST)

BLAST software is routinely used for predicting the function of a protein based on the sequences’ similarity search [48]. In this study, BLAST has been used to discriminate between the R5- and the X4-tropic sequences at E-values ranging from 10^−1^ to 10^−17^. As shown in the Table 2, maximum accuracy of 93.16% for R5-tropic and 75.75% for X4-tropic sequences at E-value cut-off 10^−3^ was achieved (Table 2, Table S4, S5).

**Table 2:** The performance of BLAST (blast-2.2.18) on CCR5 and CXCR4 dataset at different E-value cut-offs.

Type	E-value	Total Sequences	Total Hits	No Hits	Correct Hits	Percent coverage	Percent of correct prediction
**CCR5**	10^−1^	1799	1798	1	1676	93.16	93.21
	10^−3^	1799	1798	1	1676	**93.16**	93.21
	10^−8^	1799	1796	2	1675	93.11	93.26
	10^−12^	1799	1776	23	1659	92.22	93.41
	10^−16^	1799	269	1530	245	13.62	91.01
**CXCR4**	10^−1^	598	598	0	453	75.75	75.75
	10^−3^	598	598	0	453	**75.75**	75.75
	10^−8^	598	591	7	447	74.75	75.63
	10^−12^	598	534	64	418	69.90	78.28
	10^−16^	598	50	548	41	6.86	82.00

### Hybrid Approach using BLAST and SVM Model

**Figure 3:**
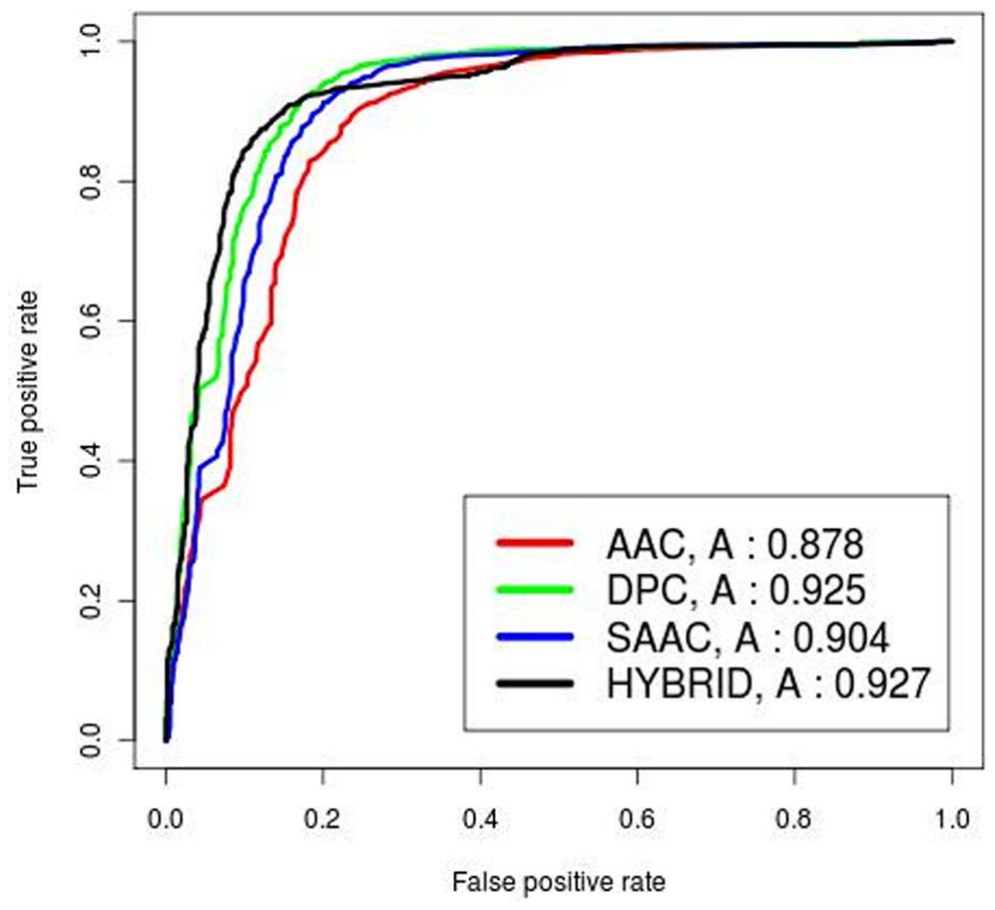
The ROC plots of four SVM models. Performance of four SVM modules (AAC, DPC, SAAC, Hybrid) by the receiver operating characteristic (ROC) plot. In the graph, ‘A’ signifies the ‘AUC’ value of the respective model.

We developed a Hybrid approach by combining SAAC based SVM model and the similarity based BLAST search. Using Hybrid approach, 91.66% sensitivity, 81.77% specificity, and 89.19% accuracy with MCC value of 0.72 was achieved (see Table S6). The Receiver Operating Curve (ROC) curves were plotted using the ROCR package [49]. The performance of the various models is shown in Figure 3.

### Sequence Analysis by WebLogo and Two Sample Logo (TSL)

**Figure 4:**
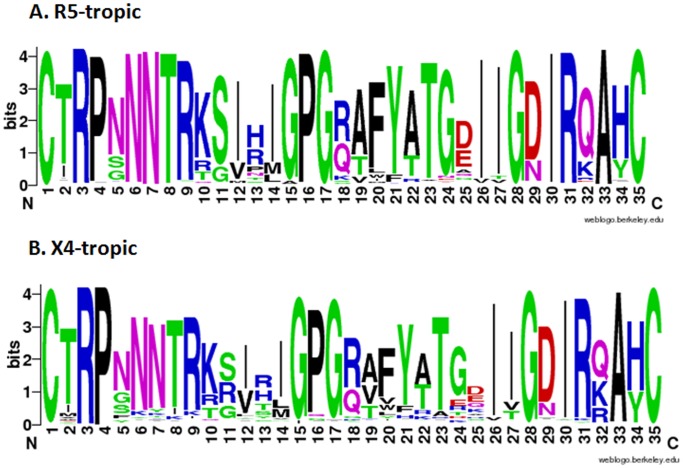
Sequence logos of R5-tropic (N = 1525) and X4-tropic (N = 408) V3 sequences. The overall height of the stack indicates the sequences conservation at the specific site, while the height of the symbols within the stack indicates the relative frequency of each amino acid at the specific site. 'N' denotes the number of sequences used in the sequence logos.

Sequence logo represents the relative amino acid frequencies at each position in a set of peptides/proteins of fixed length; it is primarily used to identify the highly conserved positions [50]. The R5- and X4-tropic sequence logos showed the similarities (*e.g*. Cysteine residue at terminal positions) and the differences (*e.g*. amino acid relative frequencies at positions 11/25) between the two types of sequences, which is clearly visible in the sequence logos (Figure 4).

**Figure 5:**
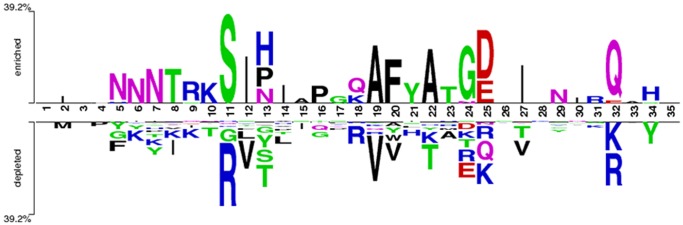
The two sample logo of R5- (N = 1525) and X4-tropic (N = 408) V3 sequences. The residues with significant difference in the frequency in two datasets are prominent at the specific sites. The positions with no residues are those where the frequency of an amino acid was approximately equal in two datasets.

Two sample logos represent the relative frequencies of amino acids at a position in the two datasets (R5-tropic as the positive sample and X4-tropic as the negative sample) [51]. In two-sample logo, the sites with no residues are those having equal frequencies of amino acids, thereby resulting in the relative frequency of ‘Zero’ *e.g.* Cysteine at position 1 and 35 in both the datasets. As stated by 11/25 rule, the relative frequency of the positively charged amino acids (*e.g.* Arginine and Lysine) were found to be highest at 11^th^ and 25^th^ positions in the X4-tropic sequences, evident from TSL (Figure 5).

### Two Sample Logo based SVM Model

TSL also provides output format as TXT (raw values), which have frequency of residues where significant differences exist between the positive and the negative samples (Table S26). Using these values, the residue frequencies in CCR5 and CXCR4 datasets were calculated by perl script. A SVM model was developed using residue frequencies at each position of the V3 sequences and achieved maximum accuracy of 88.20% (Table 3, Table S7).

**Table 3:** The performance of various SVM models developed by using Binary, TSL and the combination of Binary and TSL based input vectors.

Method	Threshold	Sensitivity	Specificity	Accuracy	MCC
**TSL**	0.4	92.07	73.77	88.20	0.65
**Binary**	0.3	92.98	78.19	89.86	0.70
**Binary+TSL**	0.4	94.36	75.00	90.27	0.70

### Binary Patterns Based SVM Model

We generated binary patterns for V3 sequences where each position of the V3 sequence was represented by a binary vector of dimension 20 [52]. Thus, the V3 sequence containing 35 amino acids is represented by a binary pattern of dimension 700 (35 × 20). The binary based SVM model has achieved maximum accuracy of 89.86% with 92.98% sensitivity, 78.19% specificity and MCC 0.70 (Table 3, Table S8).

### Two Sample Logo and Binary Based SVM Model

**Figure 6:**
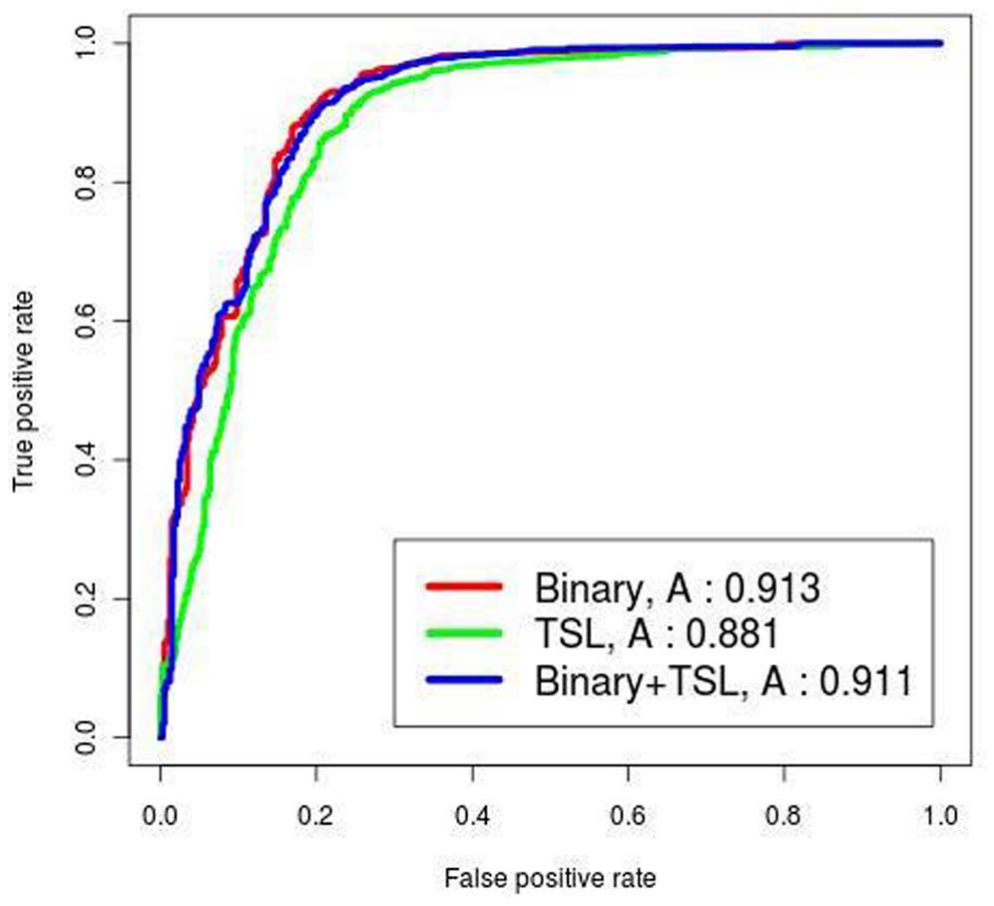
The ROC plot of Binary, TSL and Binary+TSL based SVM models. Performance of discrimination between the R5**-** and X4**-**tropic sequences by three SVM modules in the ROC plot. In the graph, ‘A’ signifies the ‘AUC’ value of the respective models.

It has been shown in the past that combination of features may achieve better accuracy [47]. Thus, we developed a SVM based model using Binary and TSL features and achieved 94.36% sensitivity, 75.00% specificity, 90.27% accuracy and 0.70 MCC (Table 3, Table S9). In order to assess the overall performance of SVM based models developed using Binary, TSL and Binary+TSL as features sets, we computed the performance of the models in term of Area Under Curve (AUC) using ROCR package. The performance of each SVM based model is shown by ROC curves (Figure 6).

### Performance on Independent Dataset

We have evaluated the performance of our SAAC and Hybrid models on an independent dataset. It was observed that both these models performed reasonably well on the independent dataset. SAAC as well as Hybrid approach based SVM models achieved accuracy 84.87% with 0.63 MCC (Table 4, Table S10, S11).

**Table 4:** The performance of SAAC and Hybrid approach on the independent dataset.

Method	Threshold	Sensitivity	Specificity	Accuracy	MCC
SAAC	0.3	85.55	82.72	84.87	0.63
Hybrid	0.3	85.55	82.72	84.87	0.63

### Comparison with Existing Methods

We evaluated the performance of our approaches (SAAC and Hybrid models) on datasets used in the previous studies [27], [29], [30], [32], [36]. The performance of our models have been compared to the performance of five methods on their datasets, WetCat [27], CPSSM [29], dskernel [32], Dybowski’s method [36], Xu’s method [30] (Table 5). As shown in Table 5, our models have better accuracy and specificity than WetCat. In case of CPSSM, our models showed comparable performance where sensitivity was lower but specificity was higher. We have compared our approach with three models of dskernel method. In case of CCR5, the training and testing were carried out on the same dataset as used by the authors. Our models showed better specificity than dskernel-R5 while the accuracy and the sensitivity remained comparable. In comparison to dskernel-X4 and dskernel-R5X4, our models have slightly better performance than these two models. When compared the performance with Dybowski’s method, it was found that our models had nearly same specificity (R5 prediction) but achieved significantly higher sensitivity. In case of Xu’s method, our approaches have achieved similar or better sensitivity (98.76% and 99.17%); the specificity was higher in case of Hybrid approach. It is clear from the above analysis that SAAC as well as Hybrid approaches are capable of predicting CCR5 as well as CXCR4 usage with high accuracy, when compared with the earlier methods on their original datasets. It is important to mention that we considered the best possible E-value in hybrid approach while comparing with other methods (Table 5, Table S12, S13, S14, S15, S16, S17, S18, S19, S20, S21, S22, S23, S24, S25).

**Table 5:** The performance and comparison of our models SAAC and Hybrid on the datasets used in previous studies.

Details of datasets	Method/model	Sensitivity	Specificity	Accuracy	MCC
Pillai *et al.*, [27], (R5–168, X4–103)	WetCat	97.6	75.7	90.86	-
	**SAAC**	**97.62**	**86.41**	**93.36**	**0.86**
	**Hybrid**	**96.43**	**87.38**	**92.99**	**0.85**
Jensen *et al.*, [29] (R5–228, X4–51)	CPSSM	75*	94	–	–
	**SAAC**	**72.55**	**94.74**	**90.68**	**0.68**
	**Hybrid**	**74.51**	**96.49**	**92.47**	**0.74**
Boisvert *et al.*, [32] (Train-1425, Test-1425)	dskernel-R5	98.75	83.55	96.35	–
	**SAAC**	**98.42**	**91.11**	**97.26**	**0.90**
	**Hybrid**	**95.50**	**95.11**	**95.44**	**0.85**
Boisvert *et al.*, [32] (Train-1425, Test-1425)	dskernel-X4	87.68	97.56	94.80	–
	**SAAC**	**89.70**	**97.08**	**95.02**	**0.88**
	**Hybrid**	**91.46**	**98.34**	**96.42**	**0.91**
Boisvert *et al.*, [32] (Train-1425, Test-1425)	dskernel-R5X4	65.89	99.20	95.15	–
	**SAAC**	**69.94**	**98.48**	**95.02**	**0.75**
	**Hybrid**	**65.90**	**99.36**	**95.30**	**0.76**
Dybowski *et al.*, [36] (R5–1151, X4–166)		81*	97	–	–
	**SAAC**	**89.16**	**98.70**	**97.49**	**0.89**
	**Hybrid**	**94.58**	**99.65**	**99.01**	**0.95**
Xu *et al.*, [30] (Train-1516, Test-642)		98.4	85.2	95.1	0.87
	**SAAC**	**98.76**	**87.34**	**95.95**	**0.89**
	**Hybrid**	**99.17**	**90.51**	**97.04**	**0.92**

* signify that in case of Jensen *et al*. [29] and Dybowski *et al*. [36] the Sensitivity refers to the ‘CXCR4’ prediction, whereas in other studies it denotes the ‘CCR5’ prediction.

### Prediction of X4 Usage by R5X4-tropic Sequences

It has been previously reported that the bioinformatics programs underestimated the frequency of CXCR4 usage by R5X4-tropic HIV-1 in brain and other tissues. To know the coreceptor usage of 30 R5X4-tropic sequences, we used the same set of sequences as used by Mefford *et al*. [53]. Before the coreceptor usage analysis, the V3 sequences were generated as full-length by replacing gaps (−) with the consensus residue. It is important to mention that the accuracy achieved by ‘HIVcoPred’ method is equal to that of SVMgeno2pheno, *i.e.* 90% (27/30), which is the highest among the seven methods tested in that study.

### Coreceptor Usage Prediction of HIV-1 Subtype A/D Sequences

We have tested the performance of HIVcoPred on 61 unique sequences of subtype D, originally used by Huang *et al*. [54]. First, the given V3 sequences were regenerated by replacing the dots (.) with consensus residues, keeping the mutated residues intact in the sequences. The gaps were also removed before the final prediction of the coreceptor usage. It was found that HIVcoPred achieved the highest ‘overall concordance’ of 65.57% in comparison to the two approaches used by Huang *et al*., *i.e.* 11/25 rule and PSSM (Table 6).

**Table 6:** Comparison of the performance of HIVcoPred with other methods on subtype D V3 sequences (N = 61), originally used by Huang *et al*. [54].

Method	Overallconcordance	SenX4	SenR5	SpX4	SpR5X4
**11/25 rule**	61%	44%	74%	71.42%	16.67%
**PSSM**	59%	67%	53%	100%	30.76%
**HIVcoPred**	65.57%	96.29%	41.17%	100%	92.30%

**SenX4** - Proportion of all viruses that could use X4 and were predicted to be X4-tropic; **SenR5** - Proportion of all viruses that only use CCR5 that were predicted to be R5-tropic; **SpX4** - correctly predicted CXCR4 usage for all the X4-tropic clones; **SpR5X4** - correctly predicted CXCR4 usage for all the dual-tropic clones; ‘N’ is the number of V3 sequences used for this analysis.

We also compared the performance of HIVcoPred method with the ‘geno2pheno’ and ‘subtype B combined rule’ on 26 subtype D sequences [55]. It was found that like these two approaches, our approach also predicted with 100% accuracy for the subtype D CXCR4 usage. The Hybrid approach has achieved 86.36% specificity whereas the specificity achieved by ‘geno2pheno10’ and ‘combined 11/25 and net charge rule’ were 54% and 68%, respectively (Table 7).

**Table 7:** Comparison of HIVcoPred with other methods on 26 V3 sequences of HIV-1 subtype D, the sequences originally used by Raymond *et al*. [55].

Method	Correctly predicted X4/No. of X4	Correctly predicted R5/No. of R5	Sensitivity	Specificity	Accuracy
**Geno2pheno10**	4/4	12/12	100%	54%	61.53%
**SubtypeB combined rule**	4/4	15/22	100%	68%	73.07%
**HIVcoPred**	4/4	19/22	100%	86.36%	88.46%

## Discussion

For any anti-HIV drug targeting CCR5 receptor, it is mandatory to know the exact type of coreceptor used by the infecting virus. Consequently, for the drug Maraviroc, which acts as a CCR5 antagonist, knowledge of the coreceptor used by HIV strains is a prerequisite [11], [12]. In the past, various genotypic as well as phenotypic methods have been developed to elucidate the coreceptor used by HIV-1 [25]–[36]. The prediction accuracy of the genotypic methods is high for R5-usage but relatively poor for the X4-usage prediction. The performance of the previously developed prediction methods ranged from sensitivity (X4) of 0.69 to 0.80, specificity (R5) of 0.93 to 0.98 and accuracy of 0.90 to 0.92 [27], [31], [33], [34], [35]. The possible reasons for the poor prediction of X4-tropism could be – (1) The unavailability of a large number of X4-tropic sequences required during the training of the models and/or (2) The high level of variation (mutations) in the X4-tropic sequences, leading to the poor training of the models. Consequently, there is a pressing need for new methods, which can predict the coreceptor usage with high accuracy.

In the present study, a method has been developed using various features, *e.g.* AAC, DPC, SAAC, *etc.,* which predicts CCR5 and CXCR4 coreceptor usage with high accuracy. For the development of this method, a well-accepted machine learning technique ‘SVM’ has been employed. SVM has been previously used in the development of various methods pertaining to the coreceptor usage prediction [27], [31], [32], [35]. It has been observed that the composition differences exist between the two types of V3 sequences, *e.g.* more Asparagine in R5-tropic and Lysine, Arginine and Tryptophan in X4-tropic sequences (Figure 1). We analysed the physico-chemical properties of the amino acid residues, and it was noticed that the X4-tropic sequences have more charged residues (mostly positively charged and large amino acids) in comparison to the R5-tropic sequences which have primarily negatively charged and small amino acid residues (Figure 2). This difference is important as the overall (net) charge changes from negative towards positive in R5- to X4-tropic sequences, corroborating the fact that the amino acids’ change affects the coreceptor tropism [56]. It is well known that the V3 sequences generally have ‘Cysteine’ at both the terminals and the crown motif (*e.g.* GPGR) in the centre of the sequence. We analysed the N- and C-terminal residues of the R5- and X4-tropic sequences. It was revealed that at N-terminal (17 residues) Asparagine, Proline, Serine were more abundant in R5-tropic whereas Lysine, Arginine, Tyrosine were abundant in X4-tropic sequences. At the C-terminal (18 residues), amino acids Alanine, Aspartic acid, Phenylalanine, Isoleucine and Glutamine were more abundant in R5-tropic whereas Lysine, Arginine and Valine were more abundant in X4-tropic V3 sequences (Figure S1, S2).

The sequence logos clearly showed similarity between R5- and X4-tropic sequences, *e.g*. presence of ‘Cysteine’ at terminus and central motif ‘GPGR’. The differences in residue frequency at the 11^th^ and the 25^th^ position are evidently visualized by the sequence logo (Figure 4). The two sample logo clearly showed that the relative frequencies of the positively charged (R/K) amino acids is more in X4- than R5-tropic sequences (Figure 5). It has been found that BLAST performed very well in identifying the R5 sequences (93.16%), but it performed relatively poor in case of X4 (75.75%). This suggests that R5-tropic sequences are more similar to other R5 sequences whereas X4-tropic is less similar to other X4 sequences in the BLAST database. This implies that simple BLAST cannot be used in the prediction of R5- and X4-tropic sequences, especially X4 sequences.

**Figure 7:**
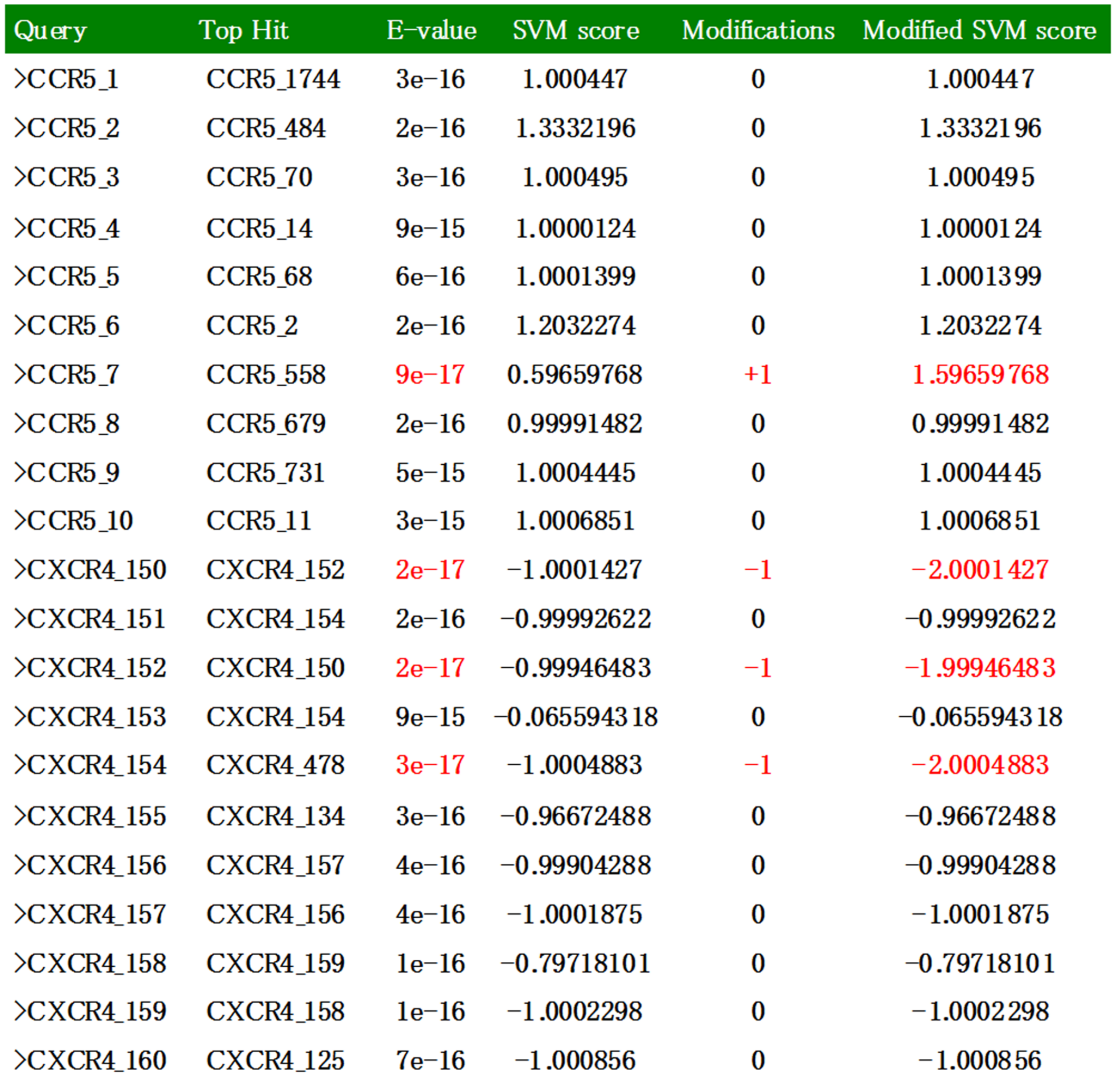
Procedure of Modified SVM scores generation by the Hybrid approach. The SVM score is first generated by SAAC based SVM model. Depending upon the top matched sequences and its E-value (in BLAST output) the SVM score has been modified by 1(+/−), which finally used in the prediction purpose.

In order to develop a model with high accuracy of the coreceptor usage prediction, we integrated our SAAC approach with BLAST to form an entirely new ‘Hybrid’ approach. The SAAC based SVM score of a given sequence was modified depending upon the BLAST hits of that particular sequence. The modified SVM score was used for the final prediction of coreceptor usage (Figure 7). As shown in Table 5, our approaches ‘SAAC’ as well as ‘Hybrid’ perform reasonably well on the dataset of earlier published studies. Moreover, It has been found that our approaches performed reasonably well when applied on the independent dataset (Table 4).

It is important to mention the advantages of any newly developed method. Since we have trained our models on a large dataset containing sequences of all the subtypes of HIV-1, it is efficient in predicting the coreceptor for all the subtypes of HIV-1, except subtype ‘O’. Previous reports suggested that the bioinformatics programs perform poorly in case of HIV-1 subtype ‘O’ [57]. This is due to the non-availability of the subtype ‘O’ sequences required in the training of various prediction models. It has been reported that the CXCR4 coreceptor usage in R5X4-tropic sequences is underestimated; our method predicted correctly and achieved accuracy up to 90%, which is equal to the highest performance reported by Mefford *et al*. [53]. It has been observed that on 61 unique sequences of HIV-1 subtype D, our method achieved the ‘highest concordance’, in comparison to 11/25 rule and PSSM (6). It has also been observed that ‘HIVcoPred’ method achieved the highest accuracy in CXCR4 as well as CCR5 coreceptor usage prediction for HIV-1 subtype D sequences (Table 7). In short, it can be said that ‘HIVcoPred’ is an efficient method for coreceptor usage predictions, not only for HIV-1 subtype B but also for non-B subtypes. We anticipate that the webserver ‘HIVcoPred’ would be highly useful in interpreting the coreceptor usage and successful management of HIV-1 infected patients.

### Conclusion

Knowing that the coreceptor usage determination is vital before starting the CCR5-antagonist based regime, the accurate prediction of the coreceptor usage is of high importance. Various genotypic methods predicted the CCR5 coreceptor usage with high accuracy, but poor in case of CXCR4. In this study, we have tested various approaches and found that the Hybrid (SAAC+BLAST) approach is highly accurate in predicting the R5- as well as the X4-tropic sequences. A SVM based model was developed using this technique and integrated into coreceptor usage prediction webserver. This webserver will be helpful in the prediction of R5- as well as X4-tropic sequences with high accuracy. The webserver ‘HIVcoPred’ is freely available at http://www.imtech.res.in/raghava/hivcopred.

## Methods

### Datasets

We extracted 5181 R5, 1018 R5X4- and 612 X4-tropic V3 sequences of HIV-1 from the Los Alamos HIV sequence database (http://www.hiv.lanl.gov/). After removing all the duplicate sequences, finally we got 1799 R5, 352 R5X4- and 246 X4-tropic unique V3 sequences. We merged the R5X4 sequences into X4-tropic dataset to form a dataset of 598 sequences. Out of these, 1525 R5- and 408 X4-tropic V3 sequences have 35 amino acids. In summary, our main dataset have 1799 R5- and 598 X4-tropic unique V3 sequences where no two sequences were identical (http://www.imtech.res.in/raghava/hivcopred/suppliment.html).

### Independent Dataset

It is important to evaluate the performance of a newly developed method on an independent dataset. For independent dataset, we extracted all the V3 sequences used in the previous studies [27], [29], [32], [36] and removed the sequences that were common with our main dataset (1799/598). We have also removed any possible identical sequences from remaining V3 sequences. In this way, we obtained an independent dataset containing 256 R5- and 81 X4-tropic V3 sequences.

### Support Vector Machine

In this study, we have employed a highly successful machine learning technique known as “Support Vector Machine” which is freely available at http://www.cs.cornell.edu/People/tj/svm_light/, version SVM-light V6.01. SVM is based on the structural risk minimization principle of statistics learning theory [58]. It is a set of related supervised learning methods used for classification and regression purposes. Users can choose a number of parameters and kernels in SVM (*e.g.* linear, polynomial, radial and sigmoid) or any user-defined kernel. The complete detail of SVM can be obtained from Vapnik, 1995 [59].

### Compositions Patterns

The general length of V3 sequence is 35 amino acids, but it may vary from 31 to 39. The aim of calculating the composition of V3 sequence is to convert the variable length of the sequences to the fixed length vectors. This is important and a crucial step because SVM requires definite length numerical vectors as input. The AAC is the fraction of each amino acid in a V3 sequence and provides a vector of 20 dimensions. The DPC was used to encapsulate the global information about each V3 sequence, which gives a fixed length pattern of 400 (20 × 20) dimensions of vector. In the case of SAAC, a sequence was divided into non-overlapping fragments and amino acid composition of each fragment was calculated independently [60], [61]. Thus, the dimension of the final input vector was N × 20, where N is the number of fragments. In this study, V3 sequences were divided into two parts (N = 2) generating 40 input dimensions, respectively. All these input vectors have been used to develop SVM models.

### Binary Patterns Generation

It has been shown in previous studies that the binary patterns of presenting amino acids in a protein result in good prediction methods [62]. The peptide of length N was represented by a vector of dimension N × 20, where each residue is represented by a vector of 20 dimensions (*e.g.* Ala by 1,0,0,0,0,0,0,0,0,0,0,0,0,0,0,0,0,0,0,0,0; Cys by 0,1,0,0,0,0,0,0,0,0,0,0,0,0,0,0,0,0,0,0,0); contains 20 amino acids. Considering V3 peptide length of 35 only, input vector of 700 dimensions were generated and used as input variables for SVM model generation and classification purpose.

### TSL Matrix Based Input Vectors

TSL is an online tool (http://www.twosamplelogo.org/cgi-bin/tsl/tsl.cgi) which distinguishes the residue frequency between two types of datasets, on each of the positions of the given sample sequences. Besides generating a graphical representation of the two given datasets (Positive and Negative sample), it also generates the output format as TXT (raw values) which is the residues’ frequency difference in the two samples with significance value (as shown by p-value). This table with position-specific frequency value was used to generate the frequency score of residues in CCR5 and CXCR4 sequences independently. Since each V3 peptide was 35 amino acids long, so an input vector of 35 dimensions was generated and used as an input vector for the SVM model generation (Table S26).

### Basic Local Alignment Search Tool

In this study, we have used BLAST (blast-2.2.18) for predicting the R5- and X4-tropic sequences against the database (1799 R5-tropic and 598 X4-tropic sequences), using ‘blastpgp’ program at E-value cut-off 0.001. Using the same set of sequences (1799/598) as the query, leaving the top self-hit, we have calculated the performance of BLAST in terms of accuracy (percentage coverage) as well as the percent of correct prediction. The number of positive and negative sequences not having any hit (target) is considered as false negative and false positive, respectively.

### Hybrid Approach (SAAC+ BLAST)

In this study, we have introduced an entirely different approach for predicting coreceptor usage by integrating the best SVM model (SAAC) with BLAST. In this Hybrid approach, prediction was carried out done in four steps: (i) SAAC based SVM score was calculated by the model; (ii) BLAST of this sequence was done against the main database (1799/598) and recorded the E-value of sequence with maximum similarity; (iii) SVM score, and the E-value of the same sequence were analyzed and (iv) depending upon the E-value of the BLAST output; SVM score was modified in the following two ways: If the ‘top matched sequence’ was CCR5 and the E-value was “−17 or less *e.g.* −18, −19,” SVM score was modified by adding “1” in it. Similarly, if the ‘top matched sequence’ was CXCR4 and the E-value was “−17 or less *e.g.* −18, −19,” SVM score was modified by subtracting “1” from it. This was a unique way to combine the features of both SAAC based SVM model and BLAST. The final score was used to predict the status of the query sequence. In this way, the best of both the approaches have been integrated into a single output which was used in the prediction purpose (Figure 7).

### Five-fold Cross Validation

There are three main frequently used cross-validation techniques – (1) single independent dataset test (2) sub-sampling test (*e.g.* 5- or 10-fold cross validation) and (3) jackknife test or Leave One Out Cross-validation technique. These tests are widely used for examining the accuracy of any new statistical prediction method [63], [64]. In our study, we used 5-fold cross validation technique, where five sets constructed randomly from the data, one set was used for testing, and the remaining sets were used for training. This process was repeated five times in such a way that each test set was used once for testing [65], [66]. The final performance was average of the performances of five sets.

### Evaluation Parameters

The evaluation of performance of a method was done by calculating the sensitivity, specificity, accuracy and MCC of the prediction, which were routinely used in similar types of studies [67]. These parameters can be calculated by using following equations:
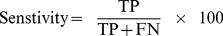
(1)

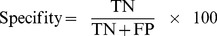
(2)

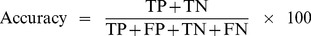
(3)

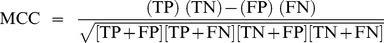
(4)Where TP is correctly predicted positive (R5-tropic) sequences, TN is correctly predicted negative (X4-tropic) sequences; FP is wrongly predicted positive (R5-tropic) sequences, and FN is wrongly predicted negative (X4-tropic) sequences.

The performance of a method is an average of the five subsets, created by five-fold cross validation technique. For evaluation of any prediction method, MCC is considered as the most robust parameter [68]. The MCC value ‘1’ corresponds to the perfect prediction, whereas ‘0’ points to a completely random prediction. The limitations of all above-described parameters are that they are threshold-dependent and they require proper optimization for the better performance. We have manually optimized all these parameters and selected the one which gave the best performance. A known threshold independent parameter is Receiver Operating Curve, which is a plot between the true positive (TP/TP+FN) proportion and false-positive (FP/FP+TN) proportion. We have used the ROCR package to plot ROC and calculating the AUC.

## Supporting Information

Figure S1 **Amino acid composition of N-terminal (17 residues) in 1799 R5- and 598 X4- tropic V3 sequences.**
(TIF)

Figure S2 **Amino acid composition of C-terminal (18 residues) in 1799 R5- and 598 X4- tropic V3 sequences.**
(TIF)

Table S1 The performance of SVM model (Learning Parameter: −z c –t 2–g 0.05–c 1–j 1) using Amino acid composition method.
(DOC)

Table S2 The performance of SVM model (Learning Parameter: −z c –t 2–g 0.01–c 3–j 1) using Dipeptide composition method.
(DOC)

Table S3 The performance of SVM model (Learning Parameter: −z c –t 2–g 0.01–c 1–j 1) using Split Amino Acid composition method.
(DOC)

Table S4 Performance of BLAST on CCR5 dataset of 1799 V3 sequences at different E-values cut-off.
(DOC)

Table S5 Performance of BLAST on CXCR4 dataset of 598 V3 sequences at different E-values cut-off.
(DOC)

Table S6 The performance of SVM model using Hybrid method.
(DOC)

Table S7 The performance of SVM model (Learning Parameter: −z c –t 2–g 15 −c 2 −j 1) using Two Sample Logo based method.
(DOC)

Table S8 The performance of SVM model (Learning Parameter: −z c –t 2–g 0.1–c 7–j 1) using Binary composition method.
(DOC)

Table S9 The performance of SVM model (Learning Parameter: −z c –t 2–g 0.1 −c 2 −j 1) using TSL+Binary composition method.
(DOC)

Table S10 The performance of Split Amino Acid Composition model on independent dataset.
(DOC)

Table S11 The performance of Hybrid model on independent dataset.
(DOC)

Table S12 The performance of SVM model (Learning Parameter: −z c –t 2–g 0.001 −c 3–j 1) based on Split Amino Acid Composition, on Pillai et al. [27] i.e. WetCat dataset.
(DOC)

Table S13 The performance of Hybrid approach on Pillai et al. [27] i.e. WetCat dataset. The E-value “≤10^−15^” was used to generate the modified SVM score by Hybrid approach.
(DOC)

Table S14 The performance of SVM model (Learning Parameter: −z c –t 2–g 0.005 −c 6–j 1) based on Split Amino Acid Composition, on Jensen et al [29] i.e. CPSSM dataset.
(DOC)

Table S15 The performance of Hybrid approach on Jensen et al. [29] i.e. CPSSM dataset. The E-value “≤10^−15^” was used to generate the modified SVM score by Hybrid approach.
(DOC)

Table S16 The performance of SVM model (Learning Parameter: −z c –t 2–g 0.005 −c 3–j 1) based on Split Amino Acid Composition, on dskenel-R5 dataset.
(DOC)

Table S17 The performance of Hybrid approach on Boisvert et al. [32] i.e. dskernel-R5 method dataset. The E-value “≤10^−17^” was used to generate the modified SVM score by Hybrid approach.
(DOC)

Table S18 The performance of SVM model (Learning Parameter: −z c –t 2–g 0.005 −c 7–j 1) based on Split Amino Acid Composition, on dskenel-X4 dataset.
(DOC)

Table S19 The performance of Hybrid approach on Boisvert et al. [32] i.e. dskernel-X4 method dataset. The E-value “≤10^−17^” was used to generate the modified SVM score by Hybrid approach.
(DOC)

Table S20 The performance of SVM model (Learning Parameter: −z c –t 2–g 0.01 −c 4–j 1) based on Split Amino Acid Composition, on dskenel-R5X4 dataset.
(DOC)

Table S21 The performance of Hybrid approach on Boisvert et al. [32] i.e. dskernel-R5X4 method dataset. The E-value “≤10^−16^” was used to generate the modified SVM score by Hybrid approach.
(DOC)

Table S22 The performance of SVM model (Learning Parameter: −z c –t 2–g 0.001 −c 7–j 1) based on Split Amino Acid Composition, on Dybowski et al. [36] dataset. This table was prepared using 10-fold cross-validation technique, as used by Dybowski et al.
(DOC)

Table S23 The performance of Hybrid approach on Dybowski et al. [36] dataset. The E-value “≤10^−15^” was used to generate the modified SVM score by Hybrid approach.
(DOC)

Table S24 The performance of SVM model (Learning Parameter: −z c –t 2–g 0.005 −c 8–j 1) based on Split Amino Acid Composition, on Xu et al. [30] dataset.
(DOC)

Table S25 The performance of Hybrid approach on Xu et al. [30] dataset. The E-value “≤10^−17^” was used to generate the modified SVM score by Hybrid approach.
(DOC)

Table S26 The TXT (raw values) showing residue frequencies of R5- and X4-tropic sequences generated by using two-sample logo method.
(DOC)
