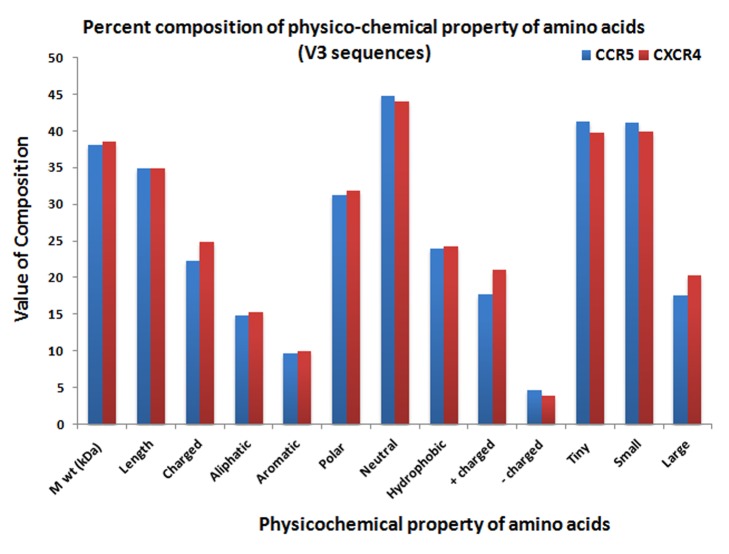# Correction: Hybrid Approach for Predicting Coreceptor Used by HIV-1 from Its V3 Loop Amino Acid Sequence

**DOI:** 10.1371/annotation/5c57dcdc-e5d9-4999-a7d0-32004427cba5

**Published:** 2013-11-07

**Authors:** Ravi Kumar, Gajendra P. S. Raghava

Figure 2 was substituted with Supplementary Figure 2. Please see the correct Figure 2 here: 

**Figure:**