# Supplementary figures and images for: Hybrid Approach for Predicting Coreceptor Used by HIV-1 from Its V3 Loop Amino Acid Sequence
Source: PLoS One. 2013 Apr 15;8(4):e61437. doi: 10.1371/journal.pone.0061437 (PMC3626595; doi:10.1371/journal.pone.0061437)

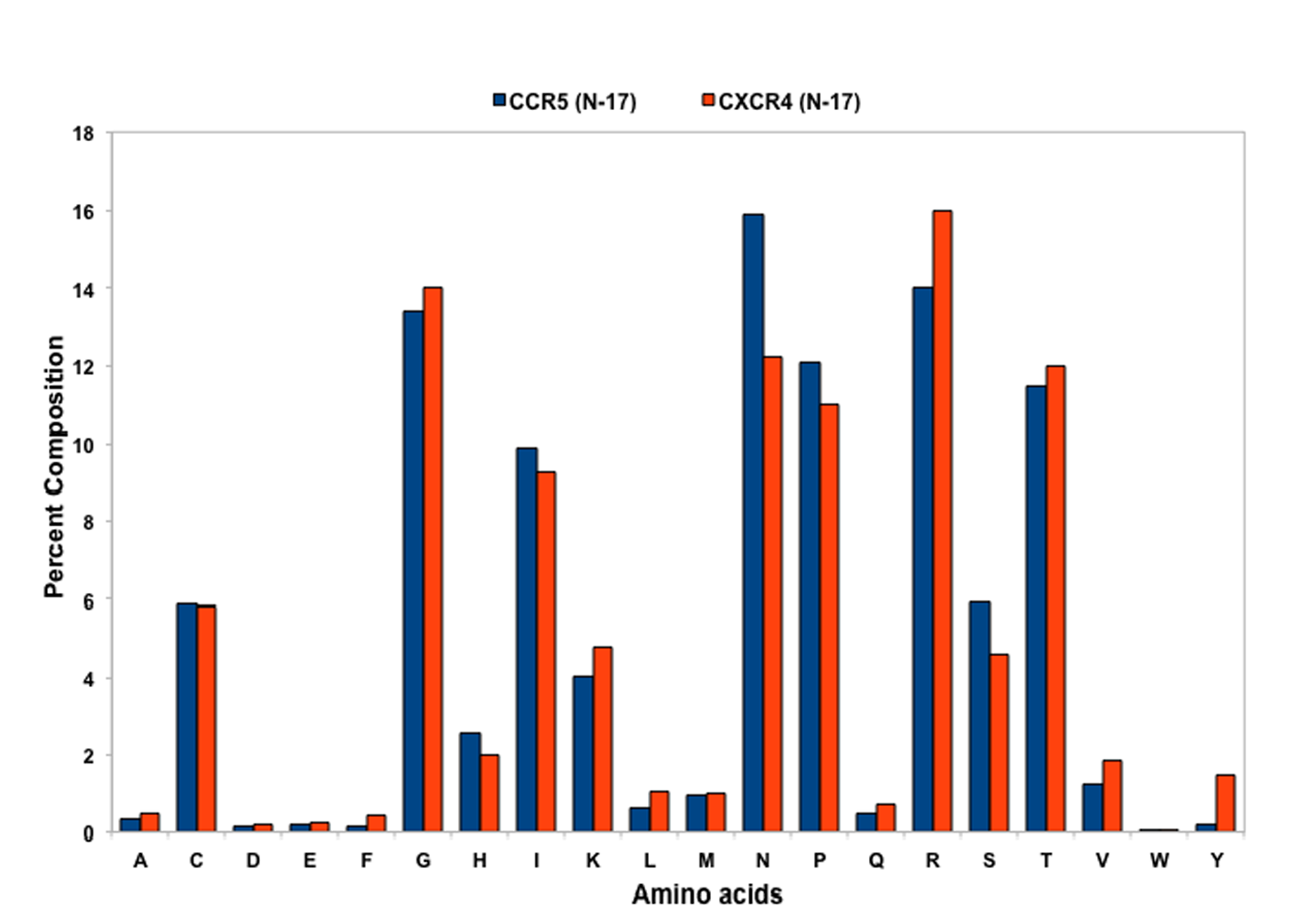

Supplement: Figure S1 — Amino acid composition of N-terminal (17 residues) in 1799 R5- and 598 X4- tropic V3 sequences. (TIF) [file pone.0061437.s001.tif]

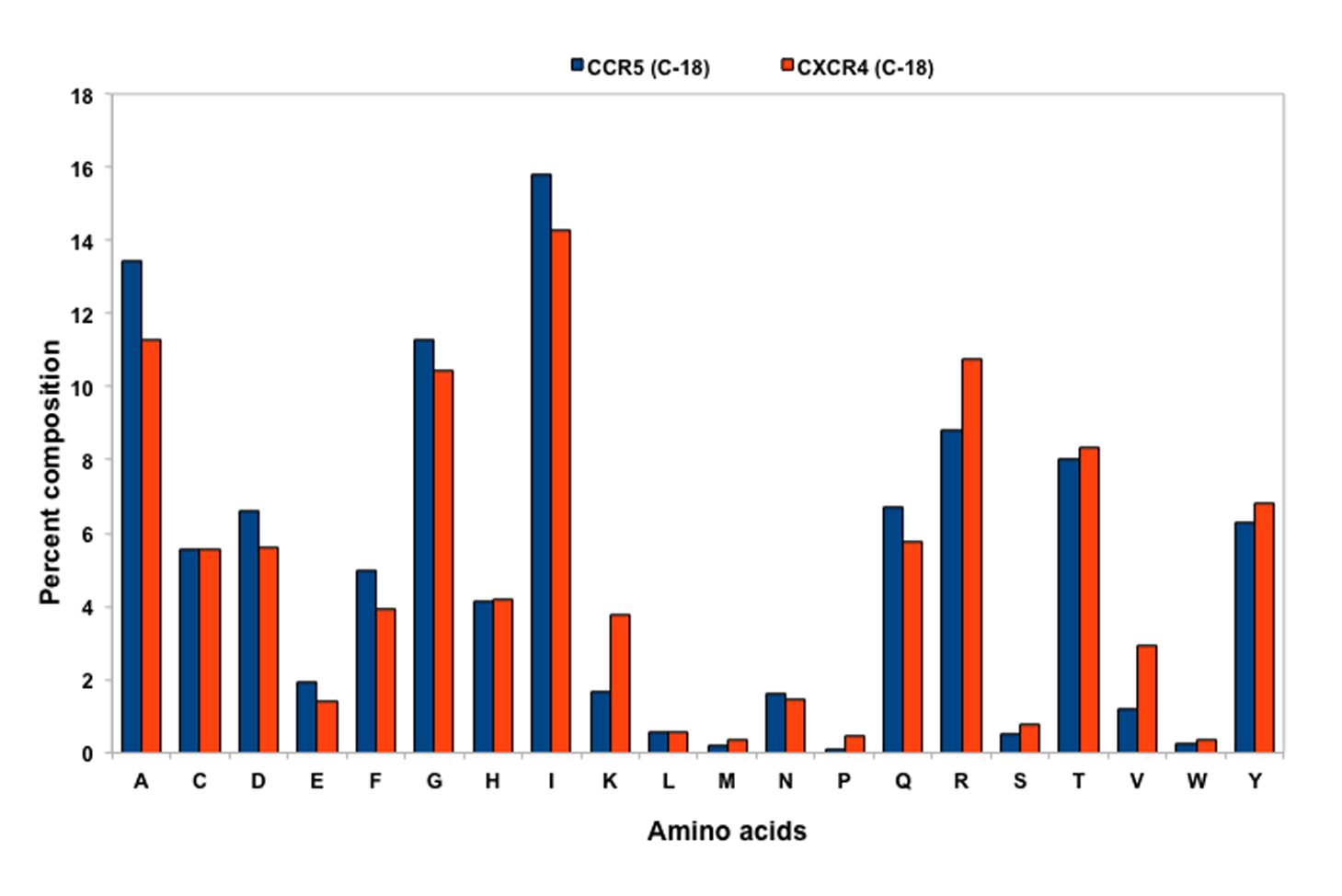

Supplement: Figure S2 — Amino acid composition of C-terminal (18 residues) in 1799 R5- and 598 X4- tropic V3 sequences. (TIF) [file pone.0061437.s002.tif]
