# Supplementary material for: Hybrid Approach for Predicting Coreceptor Used by HIV-1 from Its V3 Loop Amino Acid Sequence
Source: PLoS One. 2013 Apr 15;8(4):e61437. doi: 10.1371/journal.pone.0061437 (PMC3626595; doi:10.1371/journal.pone.0061437)
Supplement: Table S1 — The performance of SVM model (Learning Parameter: −z c –t 2–g 0.05–c 1–j 1) using Amino acid composition method. (DOC) [file pone.0061437.s003.doc]

**Table S1: The performance of SVM model (Learning Parameter: -z c –t 2 –g 0.05 –c 1 –j 1) using Amino acid composition method.**

| **Threshold** | **Sensitivity** | **Specificity** | **Accuracy** | **MCC** |
| --- | --- | --- | --- | --- |
| -1 | 100.00 | 1.84 | 75.51 | 0.12 |
| -0.9 | 99.78 | 3.68 | 75.80 | 0.14 |
| -0.8 | 99.72 | 5.69 | 76.26 | 0.18 |
| -0.7 | 99.67 | 8.70 | 76.97 | 0.24 |
| -0.6 | 99.61 | 12.54 | 77.89 | 0.29 |
| -0.5 | 99.61 | 18.06 | 79.27 | 0.36 |
| -0.4 | 99.39 | 23.58 | 80.48 | 0.41 |
| -0.3 | 99.17 | 30.94 | 82.14 | 0.47 |
| -0.2 | 98.94 | 36.62 | 83.40 | 0.51 |
| -0.1 | 98.72 | 42.31 | 84.65 | 0.56 |
| 0 | 98.33 | 47.66 | 85.69 | 0.59 |
| 0.1 | 97.55 | 54.18 | 86.73 | 0.62 |
| 0.2 | 95.16 | 65.55 | 87.78 | 0.66 |
| 0.3 | 92.16 | 71.74 | 87.07 | 0.65 |
| **0.4** | **88.77** | **76.92** | **85.82** | **0.64** |
| 0.5 | 83.10 | 81.61 | 82.73 | 0.60 |
| 0.6 | 75.26 | 83.61 | 77.35 | 0.52 |
| 0.7 | 65.93 | 86.12 | 70.96 | 0.45 |
| 0.8 | 55.92 | 88.29 | 64.00 | 0.38 |
| 0.9 | 45.69 | 91.64 | 57.15 | 0.34 |
| 1 | 35.41 | 93.81 | 49.98 | 0.28 |

(Bold value indicates the point where overall best result was achieved)
