# Supplementary material for: Hybrid Approach for Predicting Coreceptor Used by HIV-1 from Its V3 Loop Amino Acid Sequence
Source: PLoS One. 2013 Apr 15;8(4):e61437. doi: 10.1371/journal.pone.0061437 (PMC3626595; doi:10.1371/journal.pone.0061437)
Supplement: Table S2 — The performance of SVM model (Learning Parameter: −z c –t 2–g 0.01–c 3–j 1) using Dipeptide composition method. (DOC) [file pone.0061437.s004.doc]

**Table S2**: The performance of SVM model (Learning Parameter: -z c –t 2 –g 0.01 –c 3 –j 1) using Dipeptide composition method.

| **Threshold** | **Sensitivity** | **Specificity** | **Accuracy** | **MCC** |
| --- | --- | --- | --- | --- |
| -1 | 99.72 | 11.87 | 77.81 | 0.29 |
| -0.9 | 99.44 | 18.90 | 79.35 | 0.36 |
| -0.8 | 99.33 | 26.59 | 81.18 | 0.44 |
| -0.7 | 99.33 | 36.96 | 83.77 | 0.53 |
| -0.6 | 99.11 | 43.48 | 85.23 | 0.58 |
| -0.5 | 99.00 | 49.16 | 86.57 | 0.62 |
| -0.4 | 98.89 | 55.69 | 88.11 | 0.67 |
| -0.3 | 98.55 | 61.37 | 89.28 | 0.70 |
| -0.2 | 98.11 | 66.39 | 90.20 | 0.73 |
| -0.1 | 97.33 | 71.24 | 90.82 | 0.74 |
| 0 | 96.50 | 75.25 | 91.20 | 0.76 |
| 0.1 | 95.11 | 78.09 | 90.86 | 0.75 |
| **0.2** | **93.50** | **80.43** | **90.24** | **0.74** |
| 0.3 | 91.88 | 82.27 | 89.49 | 0.73 |
| 0.4 | 88.60 | 84.11 | 87.48 | 0.69 |
| 0.5 | 85.38 | 86.79 | 85.73 | 0.67 |
| 0.6 | 80.82 | 88.63 | 82.77 | 0.62 |
| 0.7 | 74.99 | 90.47 | 78.85 | 0.58 |
| 0.8 | 67.87 | 91.47 | 73.76 | 0.51 |
| 0.9 | 59.37 | 92.81 | 67.71 | 0.45 |
| 1 | 50.75 | 95.15 | 61.83 | 0.41 |

(Bold value indicates the point where overall best result was achieved)
