# Supplementary material for: Hybrid Approach for Predicting Coreceptor Used by HIV-1 from Its V3 Loop Amino Acid Sequence
Source: PLoS One. 2013 Apr 15;8(4):e61437. doi: 10.1371/journal.pone.0061437 (PMC3626595; doi:10.1371/journal.pone.0061437)
Supplement: Table S3 — The performance of SVM model (Learning Parameter: −z c –t 2–g 0.01–c 1–j 1) using Split Amino Acid composition method. (DOC) [file pone.0061437.s005.doc]

**Table S3:** The performance of SVM model (Learning Parameter: -z c –t 2 –g 0.01 –c 1 –j 1) using Split Amino Acid composition method.

| **Threshold** | **Sensitivity** | **Specificity** | **Accuracy** | **MCC** |
| --- | --- | --- | --- | --- |
| -1 | 99.94 | 2.17 | 75.55 | 0.12 |
| -0.9 | 99.83 | 4.85 | 76.14 | 0.18 |
| -0.8 | 99.67 | 10.03 | 77.30 | 0.26 |
| -0.7 | 99.61 | 14.05 | 78.26 | 0.31 |
| -0.6 | 99.56 | 19.57 | 79.60 | 0.37 |
| -0.5 | 99.50 | 26.59 | 81.31 | 0.44 |
| -0.4 | 99.44 | 33.11 | 82.90 | 0.50 |
| -0.3 | 99.39 | 39.46 | 84.44 | 0.55 |
| -0.2 | 99.17 | 45.48 | 85.77 | 0.60 |
| -0.1 | 98.78 | 49.83 | 86.57 | 0.62 |
| 0 | 98.50 | 55.35 | 87.73 | 0.65 |
| 0.1 | 97.61 | 65.72 | 89.65 | 0.71 |
| 0.2 | 94.94 | 74.58 | 89.86 | 0.72 |
| 0.3 | 92.38 | 78.60 | 88.94 | 0.71 |
| **0.4** | **88.94** | **81.44** | **87.07** | **0.67** |
| 0.5 | 84.27 | 84.45 | 84.31 | 0.63 |
| 0.6 | 77.60 | 86.62 | 79.85 | 0.57 |
| 0.7 | 68.37 | 89.13 | 73.55 | 0.50 |
| 0.8 | 58.20 | 90.97 | 66.37 | 0.43 |
| 0.9 | 48.58 | 91.97 | 59.41 | 0.36 |
| 1 | 39.69 | 93.81 | 53.19 | 0.31 |

(Bold value indicates the point where overall best result was achieved)
