# Supplementary material for: Hybrid Approach for Predicting Coreceptor Used by HIV-1 from Its V3 Loop Amino Acid Sequence
Source: PLoS One. 2013 Apr 15;8(4):e61437. doi: 10.1371/journal.pone.0061437 (PMC3626595; doi:10.1371/journal.pone.0061437)
Supplement: Table S4 — Performance of BLAST on CCR5 dataset of 1799 V3 sequences at different E-values cut-off. (DOC) [file pone.0061437.s006.doc]

**Table S4.** Performance of BLAST on CCR5 dataset of 1799 V3 sequences at different E-values cut-off.

| **E-value** | **Total Sequences** | **Total Hits** | **No Hits** | **Correct Hits** | **Percent coverage** | **Percent of correct prediction** |
| --- | --- | --- | --- | --- | --- | --- |
| 10^-1^ | 1799 | 1798 | 1 | 1676 | 93.16 | 93.21 |
| 10^-2^ | 1799 | 1798 | 1 | 1676 | 93.16 | 93.21 |
| 10^-3^ | 1799 | 1798 | 1 | 1676 | 93.16 | 93.21 |
| 10^-4^ | 1799 | 1798 | 1 | 1676 | 93.16 | 93.21 |
| 10^-5^ | 1799 | 1798 | 1 | 1676 | 93.16 | 93.21 |
| 10^-6^ | 1799 | 1796 | 2 | 1675 | 93.11 | 93.26 |
| 10^-7^ | 1799 | 1796 | 2 | 1675 | 93.11 | 93.26 |
| 10^-8^ | 1799 | 1796 | 2 | 1675 | 93.11 | 93.26 |
| 10^-9^ | 1799 | 1796 | 2 | 1675 | 93.11 | 93.26 |
| 10^-10^ | 1799 | 1795 | 4 | 1674 | 93.05 | 93.05 |
| 10^-11^ | 1799 | 1790 | 9 | 1670 | 92.83 | 93.29 |
| 10^-12^ | 1799 | 1776 | 23 | 1659 | 92.22 | 93.41 |
| 10^-13^ | 1799 | 1750 | 49 | 1637 | 90.99 | 93.54 |
| 10^-14^ | 1799 | 1677 | 122 | 1571 | 87.33 | 93.67 |
| 10^-15^ | 1799 | 1336 | 463 | 1264 | 70.26 | 94.61 |
| 10^-16^ | 1799 | 269 | 1530 | 245 | 13.62 | 91.01 |
| 10^-17^ | 1799 | 0 | 1799 | 0 | 0 | - |
