# Supplementary material for: Hybrid Approach for Predicting Coreceptor Used by HIV-1 from Its V3 Loop Amino Acid Sequence
Source: PLoS One. 2013 Apr 15;8(4):e61437. doi: 10.1371/journal.pone.0061437 (PMC3626595; doi:10.1371/journal.pone.0061437)
Supplement: Table S5 — Performance of BLAST on CXCR4 dataset of 598 V3 sequences at different E-values cut-off. (DOC) [file pone.0061437.s007.doc]

**Table S5.** Performance of BLAST on CXCR4 dataset of 598 V3 sequences at different E-values cut-off.

| **E-value** | **Total Sequences** | **Total Hits** | **No Hits** | **Correct Hits** | **Percent coverage** | **Percent of correct prediction** |
| --- | --- | --- | --- | --- | --- | --- |
| 10^-1^ | 598 | 598 | 0 | 453 | 75.75 | 75.75 |
| 10^-2^ | 598 | 598 | 0 | 453 | 75.75 | 75.75 |
| 10^-3^ | 598 | 598 | 0 | 453 | 75.75 | 75.75 |
| 10^-4^ | 598 | 598 | 0 | 453 | 75.75 | 75.75 |
| 10^-5^ | 598 | 598 | 0 | 453 | 75.75 | 75.75 |
| 10^-6^ | 598 | 598 | 0 | 453 | 75.75 | 75.75 |
| 10^-7^ | 598 | 593 | 5 | 448 | 74.92 | 75.54 |
| 10^-8^ | 598 | 591 | 7 | 447 | 74.75 | 75.63 |
| 10^-9^ | 598 | 584 | 14 | 443 | 74.08 | 75.85 |
| 10^-10^ | 598 | 568 | 30 | 434 | 72.58 | 76.40 |
| 10^-11^ | 598 | 554 | 44 | 428 | 71.57 | 77.26 |
| 10^-12^ | 598 | 534 | 64 | 418 | 69.90 | 78.28 |
| 10^-13^ | 598 | 506 | 92 | 401 | 67.06 | 79.25 |
| 10^-14^ | 598 | 442 | 156 | 361 | 60.37 | 81.67 |
| 10^-15^ | 598 | 344 | 254 | 283 | 47.32 | 82.27 |
| 10^-16^ | 598 | 50 | 548 | 41 | 6.86 | 82.00 |
| 10^-17^ | 598 | 0 | 598 | 0 | 0 | - |
