# Supplementary material for: Hybrid Approach for Predicting Coreceptor Used by HIV-1 from Its V3 Loop Amino Acid Sequence
Source: PLoS One. 2013 Apr 15;8(4):e61437. doi: 10.1371/journal.pone.0061437 (PMC3626595; doi:10.1371/journal.pone.0061437)
Supplement: Table S6 — The performance of SVM model using Hybrid method. (DOC) [file pone.0061437.s008.doc]

**Table S6:** The performance of SVM model using Hybrid [SAAC + BLAST] method.

| **Threshold** | **Sensitivity** | **Specificity** | **Accuracy** | **MCC** |
| --- | --- | --- | --- | --- |
| 1.0 | 43.47 | 95.48 | 56.45 | 0.36 |
| 0.9 | 51.14 | 94.15 | 61.87 | 0.40 |
| 0.8 | 59.70 | 92.98 | 68.00 | 0.46 |
| 0.7 | 68.87 | 91.14 | 74.43 | 0.52 |
| 0.6 | 77.54 | 89.30 | 80.48 | 0.59 |
| 0.5 | 83.82 | 87.12 | 84.65 | 0.65 |
| 0.4 | 88.27 | 84.45 | 87.32 | 0.69 |
| **0.3** | **91.66** | **81.77** | **89.19** | **0.72** |
| 0.2 | 94.22 | 77.26 | 89.99 | 0.73 |
| 0.1 | 96.83 | 68.90 | 89.86 | 0.72 |
| 0.0 | 97.89 | 57.69 | 87.86 | 0.66 |
| -0.1 | 98.33 | 51.67 | 86.69 | 0.62 |
| -0.2 | 98.61 | 47.32 | 85.82 | 0.59 |
| -0.3 | 98.94 | 41.30 | 84.56 | 0.55 |
| -0.4 | 99.11 | 34.78 | 83.06 | 0.50 |
| -0.5 | 99.11 | 28.43 | 81.48 | 0.45 |
| -0.6 | 99.33 | 21.74 | 79.97 | 0.39 |
| -0.7 | 99.39 | 15.89 | 78.56 | 0.32 |
| -0.8 | 99.44 | 11.71 | 77.56 | 0.27 |
| -0.9 | 99.56 | 6.19 | 76.26 | 0.18 |
| -1.0 | 99.56 | 3.34 | 75.55 | 0.12 |

(Bold value indicates the point where overall best result was achieved)
