# Supplementary material for: Hybrid Approach for Predicting Coreceptor Used by HIV-1 from Its V3 Loop Amino Acid Sequence
Source: PLoS One. 2013 Apr 15;8(4):e61437. doi: 10.1371/journal.pone.0061437 (PMC3626595; doi:10.1371/journal.pone.0061437)
Supplement: Table S7 — The performance of SVM model (Learning Parameter: −z c –t 2–g 15 −c 2 −j 1) using Two Sample Logo based method. (DOC) [file pone.0061437.s009.doc]

**Table S7:** The performance of SVM model (Learning Parameter: -z c –t 2 –g 15 -c 2 -j 1) using Two Sample Logo based method.

| **Threshold** | **Sensitivity** | **Specificity** | **Accuracy** | **MCC** |
| --- | --- | --- | --- | --- |
| -1 | 99.93 | 12.25 | 81.43 | 0.31 |
| -0.9 | 99.48 | 27.94 | 84.38 | 0.46 |
| -0.8 | 99.34 | 34.80 | 85.72 | 0.52 |
| -0.7 | 98.69 | 36.76 | 85.62 | 0.51 |
| -0.6 | 98.56 | 40.44 | 86.29 | 0.54 |
| -0.5 | 98.30 | 41.67 | 86.34 | 0.54 |
| -0.4 | 98.10 | 44.61 | 86.81 | 0.56 |
| -0.3 | 97.90 | 48.04 | 87.38 | 0.58 |
| -0.2 | 97.64 | 51.23 | 87.84 | 0.60 |
| -0.1 | 97.18 | 56.13 | 88.52 | 0.63 |
| 0 | 96.79 | 61.03 | 89.24 | 0.65 |
| 0.1 | 95.93 | 65.20 | 89.45 | 0.66 |
| 0.2 | 94.69 | 68.63 | 89.19 | 0.66 |
| 0.3 | 93.57 | 71.32 | 88.88 | 0.66 |
| **0.4** | **92.07** | **73.77** | **88.20** | **0.65** |
| 0.5 | 89.77 | 75.98 | 86.86 | 0.63 |
| 0.6 | 86.89 | 78.43 | 85.10 | 0.60 |
| 0.7 | 83.67 | 79.66 | 82.82 | 0.57 |
| 0.8 | 79.61 | 81.86 | 80.08 | 0.53 |
| 0.9 | 72.72 | 84.80 | 75.27 | 0.48 |
| 1 | 60.33 | 89.22 | 66.43 | 0.40 |

(Bold value indicates the point where overall best result was achieved)
