# Supplementary material for: Hybrid Approach for Predicting Coreceptor Used by HIV-1 from Its V3 Loop Amino Acid Sequence
Source: PLoS One. 2013 Apr 15;8(4):e61437. doi: 10.1371/journal.pone.0061437 (PMC3626595; doi:10.1371/journal.pone.0061437)
Supplement: Table S8 — The performance of SVM model (Learning Parameter: −z c –t 2–g 0.1–c 7–j 1) using Binary composition method. (DOC) [file pone.0061437.s010.doc]

**Table S8:** The performance of SVM model (Learning Parameter: -z c –t 2 –g 0.1 –c 7 –j 1) using Binary composition method.

| **Threshold** | **Sensitivity** | **Specificity** | **Accuracy** | **MCC** |
| --- | --- | --- | --- | --- |
| -1 | 99.74 | 20.83 | 83.08 | 0.40 |
| -0.9 | 99.34 | 26.96 | 84.07 | 0.44 |
| -0.8 | 99.28 | 33.82 | 85.46 | 0.51 |
| -0.7 | 99.21 | 42.16 | 87.17 | 0.58 |
| -0.6 | 98.89 | 47.79 | 88.10 | 0.61 |
| -0.5 | 98.75 | 52.94 | 89.08 | 0.65 |
| -0.4 | 98.56 | 55.39 | 89.45 | 0.66 |
| -0.3 | 98.30 | 61.52 | 90.53 | 0.70 |
| -0.2 | 97.84 | 64.71 | 90.84 | 0.71 |
| -0.1 | 97.25 | 67.40 | 90.95 | 0.71 |
| 0 | 96.59 | 68.87 | 90.74 | 0.71 |
| 0.1 | 95.67 | 74.02 | 91.10 | 0.72 |
| 0.2 | 93.84 | 75.25 | 89.91 | 0.70 |
| **0.3** | **92.98** | **78.19** | **89.86** | **0.70** |
| 0.4 | 90.69 | 80.39 | 88.52 | 0.68 |
| 0.5 | 88.00 | 82.84 | 86.91 | 0.65 |
| 0.6 | 84.52 | 83.82 | 84.38 | 0.61 |
| 0.7 | 80.13 | 85.29 | 81.22 | 0.56 |
| 0.8 | 74.10 | 86.52 | 76.72 | 0.51 |
| 0.9 | 67.93 | 88.97 | 72.37 | 0.47 |
| 1 | 60.66 | 91.18 | 67.10 | 0.42 |

(Bold value indicates the point where overall best result was achieved)
