# Supplementary material for: Hybrid Approach for Predicting Coreceptor Used by HIV-1 from Its V3 Loop Amino Acid Sequence
Source: PLoS One. 2013 Apr 15;8(4):e61437. doi: 10.1371/journal.pone.0061437 (PMC3626595; doi:10.1371/journal.pone.0061437)
Supplement: Table S9 — The performance of SVM model (Learning Parameter: −z c –t 2–g 0.1 −c 2 −j 1) using TSL+Binary composition method. (DOC) [file pone.0061437.s011.doc]

**Table S9:** The performance of SVM model (Learning Parameter: -z c –t 2 –g 0.1 -c 2 -j 1) using Binary+TSL composition method.

| **Threshold** | **Sensitivity** | **Specificity** | **Accuracy** | **MCC** |
| --- | --- | --- | --- | --- |
| -1 | 99.93 | 17.65 | 82.57 | 0.38 |
| -0.9 | 99.54 | 25.00 | 83.81 | 0.43 |
| -0.8 | 99.48 | 32.35 | 85.31 | 0.50 |
| -0.7 | 99.34 | 39.71 | 86.76 | 0.56 |
| -0.6 | 99.34 | 44.61 | 87.79 | 0.60 |
| -0.5 | 99.15 | 47.79 | 88.31 | 0.62 |
| -0.4 | 99.02 | 52.45 | 89.19 | 0.65 |
| -0.3 | 98.75 | 54.17 | 89.34 | 0.65 |
| -0.2 | 98.43 | 57.60 | 89.81 | 0.67 |
| -0.1 | 98.23 | 60.78 | 90.33 | 0.69 |
| 0 | 97.90 | 62.75 | 90.48 | 0.70 |
| 0.1 | 97.51 | 66.18 | 90.89 | 0.71 |
| 0.2 | 96.79 | 68.63 | 90.84 | 0.71 |
| 0.3 | 95.67 | 71.08 | 90.48 | 0.70 |
| **0.4** | **94.36** | **75.00** | **90.27** | **0.70** |
| 0.5 | 92.07 | 77.45 | 88.98 | 0.68 |
| 0.6 | 89.51 | 80.15 | 87.53 | 0.65 |
| 0.7 | 85.38 | 82.60 | 84.79 | 0.61 |
| 0.8 | 79.41 | 85.29 | 80.65 | 0.56 |
| 0.9 | 72.20 | 87.99 | 75.53 | 0.50 |
| 1 | 62.62 | 90.69 | 68.55 | 0.44 |

(Bold value indicates the point where overall best result was achieved)
