# Supplementary material for: Hybrid Approach for Predicting Coreceptor Used by HIV-1 from Its V3 Loop Amino Acid Sequence
Source: PLoS One. 2013 Apr 15;8(4):e61437. doi: 10.1371/journal.pone.0061437 (PMC3626595; doi:10.1371/journal.pone.0061437)
Supplement: Table S10 — The performance of Split Amino Acid Composition model on independent dataset. (DOC) [file pone.0061437.s012.doc]

**Table S10:** The performance of Split Amino Acid Composition (SAAC) model on Independent dataset.

| **Threshold** | **Sensitivity** | **Specificity** | **Accuracy** | **MCC** |
| --- | --- | --- | --- | --- |
| -1 | 100.00 | 2.47 | 76.56 | 0.14 |
| -0.9 | 99.22 | 3.70 | 76.26 | 0.10 |
| -0.8 | 99.22 | 3.70 | 76.26 | 0.10 |
| -0.7 | 98.83 | 6.17 | 76.56 | 0.14 |
| -0.6 | 98.83 | 9.88 | 77.45 | 0.21 |
| -0.5 | 98.83 | 12.35 | 78.04 | 0.25 |
| -0.4 | 98.83 | 17.28 | 79.23 | 0.31 |
| -0.3 | 98.05 | 22.22 | 79.82 | 0.34 |
| -0.2 | 97.66 | 28.40 | 81.01 | 0.40 |
| -0.1 | 96.88 | 34.57 | 81.90 | 0.43 |
| 0 | 96.09 | 44.44 | 83.68 | 0.50 |
| 0.1 | 92.19 | 67.90 | 86.35 | 0.62 |
| 0.2 | 87.50 | 76.54 | 84.87 | 0.61 |
| **0.3** | **85.55** | **82.72** | **84.87** | **0.63** |
| 0.4 | 83.59 | 85.19 | 83.98 | 0.63 |
| 0.5 | 79.30 | 87.65 | 81.31 | 0.59 |
| 0.6 | 76.56 | 87.65 | 79.23 | 0.56 |
| 0.7 | 72.27 | 88.89 | 76.26 | 0.53 |
| 0.8 | 68.36 | 90.12 | 73.59 | 0.50 |
| 0.9 | 62.89 | 93.83 | 70.33 | 0.48 |
| 1 | 52.34 | 93.83 | 62.31 | 0.40 |

(Bold value indicates the point where overall best result was achieved)
