# Supplementary material for: Hybrid Approach for Predicting Coreceptor Used by HIV-1 from Its V3 Loop Amino Acid Sequence
Source: PLoS One. 2013 Apr 15;8(4):e61437. doi: 10.1371/journal.pone.0061437 (PMC3626595; doi:10.1371/journal.pone.0061437)
Supplement: Table S11 — The performance of Hybrid model on independent dataset. (DOC) [file pone.0061437.s013.doc]

**Table S11:** The performance of Hybrid (SAAC+BLAST) model on Independent dataset.

| **Threshold** | **Sensitivity** | **Specificity** | **Accuracy** | **MCC** |
| --- | --- | --- | --- | --- |
| -1 | 98.83 | 2.47 | 75.67 | 0.05 |
| -0.9 | 98.83 | 3.7 | 75.96 | 0.08 |
| -0.8 | 98.83 | 3.7 | 75.96 | 0.08 |
| -0.7 | 98.44 | 6.17 | 76.26 | 0.12 |
| -0.6 | 98.44 | 9.88 | 77.15 | 0.19 |
| -0.5 | 98.44 | 12.35 | 77.74 | 0.23 |
| -0.4 | 98.44 | 17.28 | 78.93 | 0.30 |
| -0.3 | 98.05 | 22.22 | 79.82 | 0.34 |
| -0.2 | 97.66 | 28.4 | 81.01 | 0.40 |
| -0.1 | 96.88 | 34.57 | 81.9 | 0.43 |
| 0 | 96.09 | 44.44 | 83.68 | 0.50 |
| 0.1 | 92.19 | 67.9 | 86.35 | 0.62 |
| 0.2 | 87.5 | 76.54 | 84.87 | 0.61 |
| **0.3** | **85.55** | **82.72** | **84.87** | **0.63** |
| 0.4 | 83.59 | 85.19 | 83.98 | 0.63 |
| 0.5 | 79.3 | 87.65 | 81.31 | 0.59 |
| 0.6 | 76.95 | 87.65 | 79.53 | 0.57 |
| 0.7 | 72.66 | 88.89 | 76.56 | 0.53 |
| 0.8 | 69.14 | 90.12 | 74.18 | 0.51 |
| 0.9 | 64.06 | 93.83 | 71.22 | 0.49 |
| 1 | 57.81 | 93.83 | 66.47 | 0.44 |

(Bold value indicates the point where overall best result was achieved)
