# Supplementary material for: Hybrid Approach for Predicting Coreceptor Used by HIV-1 from Its V3 Loop Amino Acid Sequence
Source: PLoS One. 2013 Apr 15;8(4):e61437. doi: 10.1371/journal.pone.0061437 (PMC3626595; doi:10.1371/journal.pone.0061437)
Supplement: Table S12 — The performance of SVM model (Learning Parameter: −z c –t 2–g 0.001 −c 3–j 1) based on Split Amino Acid Composition, on Pillai et al. [27] i.e. WetCat dataset. (DOC) [file pone.0061437.s014.doc]

**Table S12**: The performance of SVM model (Learning Parameter: -z c –t 2 –g 0.001 -c 3 –j 1) based on Split Amino Acid Composition, on Pillai *et al.* [27] *i.e.* WetCat dataset.

| **Threshold** | **Sensitivity** | **Specificity** | **Accuracy** | **MCC** |
| --- | --- | --- | --- | --- |
| -1 | 100.00 | 50.49 | 81.18 | 0.62 |
| -0.9 | 100.00 | 58.25 | 84.13 | 0.68 |
| -0.8 | 100.00 | 62.14 | 85.61 | 0.71 |
| -0.7 | 99.40 | 66.99 | 87.08 | 0.74 |
| -0.6 | 99.40 | 69.90 | 88.19 | 0.76 |
| -0.5 | 99.40 | 75.73 | 90.41 | 0.80 |
| -0.4 | 99.40 | 77.67 | 91.14 | 0.82 |
| -0.3 | 99.40 | 79.61 | 91.88 | 0.83 |
| -0.2 | 98.21 | 81.55 | 91.88 | 0.83 |
| -0.1 | 98.21 | 85.44 | 93.36 | 0.86 |
| 0 | 98.21 | 86.41 | 93.73 | 0.87 |
| 0.1 | **97.62** | **86.41** | **93.36** | **0.86** |
| 0.2 | 96.43 | 87.38 | 92.99 | 0.85 |
| 0.3 | 96.43 | 88.35 | 93.36 | 0.86 |
| 0.4 | 95.24 | 89.32 | 92.99 | 0.85 |
| 0.5 | 93.45 | 90.29 | 92.25 | 0.84 |
| 0.6 | 88.69 | 90.29 | 89.30 | 0.78 |
| 0.7 | 85.12 | 90.29 | 87.08 | 0.74 |
| 0.8 | 79.17 | 92.23 | 84.13 | 0.69 |
| 0.9 | 75.00 | 94.17 | 82.29 | 0.67 |
| 1 | 68.45 | 94.17 | 78.23 | 0.61 |

(Bold value indicates the point where overall best result was achieved)
