# Supplementary material for: Hybrid Approach for Predicting Coreceptor Used by HIV-1 from Its V3 Loop Amino Acid Sequence
Source: PLoS One. 2013 Apr 15;8(4):e61437. doi: 10.1371/journal.pone.0061437 (PMC3626595; doi:10.1371/journal.pone.0061437)
Supplement: Table S13 — The performance of Hybrid approach on Pillai et al. [27] i.e. WetCat dataset. The E-value “≤10−15” was used to generate the modified SVM score by Hybrid approach. (DOC) [file pone.0061437.s015.doc]

**Table S13**: The performance of Hybrid approach on Pillai et al. [27] *i.e*. WetCat dataset. The E-value “≤ 10^-15^” was used to generate the modified SVM score by Hybrid approach.

| **Threshold** | **Sensitivity** | **Specificity** | **Accuracy** | **MCC** |
| --- | --- | --- | --- | --- |
| -1 | 99.4 | 55.34 | 82.66 | 0.65 |
| -0.9 | 99.4 | 61.17 | 84.87 | 0.69 |
| -0.8 | 99.4 | 66.99 | 87.08 | 0.74 |
| -0.7 | 99.4 | 69.9 | 88.19 | 0.76 |
| -0.6 | 98.81 | 70.87 | 88.19 | 0.76 |
| -0.5 | 98.81 | 74.76 | 89.67 | 0.79 |
| -0.4 | 98.81 | 78.64 | 91.14 | 0.82 |
| -0.3 | 98.21 | 83.5 | 92.62 | 0.84 |
| -0.2 | 97.62 | 86.41 | 93.36 | 0.86 |
| -0.1 | **96.43** | **87.38** | **92.99** | **0.85** |
| 0 | 95.24 | 88.35 | 92.62 | 0.84 |
| 0.1 | 94.05 | 89.32 | 92.25 | 0.84 |
| 0.2 | 92.86 | 90.29 | 91.88 | 0.83 |
| 0.3 | 92.86 | 92.23 | 92.62 | 0.84 |
| 0.4 | 91.67 | 92.23 | 91.88 | 0.83 |
| 0.5 | 91.07 | 92.23 | 91.51 | 0.82 |
| 0.6 | 90.48 | 92.23 | 91.14 | 0.82 |
| 0.7 | 89.88 | 93.2 | 91.14 | 0.82 |
| 0.8 | 88.69 | 93.2 | 90.41 | 0.8 |
| 0.9 | 86.9 | 93.2 | 89.3 | 0.78 |
| 1 | 86.9 | 94.17 | 89.67 | 0.79 |

(Bold value indicates the point where overall best result was achieved)
