# Supplementary material for: Hybrid Approach for Predicting Coreceptor Used by HIV-1 from Its V3 Loop Amino Acid Sequence
Source: PLoS One. 2013 Apr 15;8(4):e61437. doi: 10.1371/journal.pone.0061437 (PMC3626595; doi:10.1371/journal.pone.0061437)
Supplement: Table S14 — The performance of SVM model (Learning Parameter: −z c –t 2–g 0.005 −c 6–j 1) based on Split Amino Acid Composition, on Jensen et al [29] i.e. CPSSM dataset. (DOC) [file pone.0061437.s016.doc]

**Table S14**: The performance of SVM model (Learning Parameter: -z c –t 2 –g 0.005 -c 6 –j 1) based on Split Amino Acid Composition, on Jensen et al [29] *i.e*. CPSSM dataset.

| **Threshold** | **Sensitivity** | **Specificity** | **Accuracy** | **MCC** |
| --- | --- | --- | --- | --- |
| -1 | 98.04 | 62.28 | 68.82 | 0.47 |
| -0.9 | 84.31 | 73.68 | 75.63 | 0.46 |
| -0.8 | 84.31 | 78.95 | 79.93 | 0.52 |
| -0.7 | 84.31 | 83.33 | 83.51 | 0.58 |
| -0.6 | 82.35 | 86.4 | 85.66 | 0.6 |
| -0.5 | 80.39 | 88.16 | 86.74 | 0.62 |
| -0.4 | 78.43 | 90.35 | 88.17 | 0.64 |
| -0.3 | 78.43 | 91.67 | 89.25 | 0.66 |
| -0.2 | 76.47 | 93.42 | 90.32 | 0.68 |
| **-0.1** | **72.55** | **94.74** | **90.68** | **0.68** |
| 0 | 68.63 | 96.05 | 91.04 | 0.69 |
| 0.1 | 64.71 | 96.05 | 90.32 | 0.66 |
| 0.2 | 58.82 | 96.49 | 89.61 | 0.62 |
| 0.3 | 50.98 | 96.93 | 88.53 | 0.57 |
| 0.4 | 49.02 | 96.93 | 88.17 | 0.56 |
| 0.5 | 47.06 | 97.37 | 88.17 | 0.55 |
| 0.6 | 45.1 | 97.37 | 87.81 | 0.54 |
| 0.7 | 39.22 | 97.81 | 87.1 | 0.5 |
| 0.8 | 35.29 | 98.25 | 86.74 | 0.48 |
| 0.9 | 33.33 | 98.25 | 86.38 | 0.46 |
| 1 | 21.57 | 99.12 | 84.95 | 0.38 |

(Bold value indicates the point where overall best result was achieved)
