# Supplementary material for: Hybrid Approach for Predicting Coreceptor Used by HIV-1 from Its V3 Loop Amino Acid Sequence
Source: PLoS One. 2013 Apr 15;8(4):e61437. doi: 10.1371/journal.pone.0061437 (PMC3626595; doi:10.1371/journal.pone.0061437)
Supplement: Table S15 — The performance of Hybrid approach on Jensen et al. [29] i.e. CPSSM dataset. The E-value “≤10−15” was used to generate the modified SVM score by Hybrid approach. (DOC) [file pone.0061437.s017.doc]

**Table S15**: The performance of Hybrid approach on Jensen et al. [29] *i.e*. CPSSM dataset. The E-value “≤ 10^-15^” was used to generate the modified SVM score by Hybrid approach.

| **Threshold** | **Sensitivity** | **Specificity** | **Accuracy** | **MCC** |
| --- | --- | --- | --- | --- |
| -1 | 88.24 | 90.35 | 89.96 | 0.71 |
| -0.9 | 84.31 | 90.35 | 89.25 | 0.68 |
| -0.8 | 84.31 | 90.79 | 89.61 | 0.69 |
| -0.7 | 82.35 | 91.67 | 89.96 | 0.69 |
| -0.6 | 82.35 | 91.67 | 89.96 | 0.69 |
| -0.5 | 82.35 | 92.11 | 90.32 | 0.7 |
| -0.4 | 80.39 | 92.98 | 90.68 | 0.7 |
| -0.3 | 80.39 | 93.42 | 91.04 | 0.71 |
| -0.2 | 80.39 | 93.86 | 91.4 | 0.72 |
| -0.1 | 80.39 | 94.74 | 92.11 | 0.74 |
| **0** | **74.51** | **96.49** | **92.47** | **0.74** |
| 0.1 | 64.71 | 96.93 | 91.04 | 0.68 |
| 0.2 | 60.78 | 97.37 | 90.68 | 0.66 |
| 0.3 | 60.78 | 97.37 | 90.68 | 0.66 |
| 0.4 | 56.86 | 97.37 | 89.96 | 0.63 |
| 0.5 | 56.86 | 98.25 | 90.68 | 0.66 |
| 0.6 | 54.9 | 98.68 | 90.68 | 0.66 |
| 0.7 | 52.94 | 98.68 | 90.32 | 0.64 |
| 0.8 | 47.06 | 99.12 | 89.61 | 0.61 |
| 0.9 | 43.14 | 99.12 | 88.89 | 0.58 |
| 1 | 35.29 | 99.12 | 87.46 | 0.52 |

Please note that since CPSSM method considered X4-sequences as positive examples, the hybrid approach also calculated the modified SVM score by adding ‘1’ to the SAAC based SVM score if the top BLAST hit was CXCR4; and by subtracting ‘1’ from the SAAC based SVM score if the top BLAST hit was CCR5 sequence.
