# Supplementary material for: Hybrid Approach for Predicting Coreceptor Used by HIV-1 from Its V3 Loop Amino Acid Sequence
Source: PLoS One. 2013 Apr 15;8(4):e61437. doi: 10.1371/journal.pone.0061437 (PMC3626595; doi:10.1371/journal.pone.0061437)
Supplement: Table S16 — The performance of SVM model (Learning Parameter: −z c –t 2–g 0.005 −c 3–j 1) based on Split Amino Acid Composition, on dskenel-R5 dataset. (DOC) [file pone.0061437.s018.doc]

**Table S16**: The performance of SVM model (Learning Parameter: -z c –t 2 –g 0.005 -c 3 –j 1) based on Split Amino Acid Composition, on dskenel-R5 dataset.

| **Threshold** | **Sensitivity** | **Specificity** | **Accuracy** | **MCC** |
| --- | --- | --- | --- | --- |
| -1 | 99.92 | 25.33 | 88.14 | 0.47 |
| -0.9 | 99.92 | 58.22 | 93.33 | 0.73 |
| -0.8 | 99.92 | 60 | 93.61 | 0.74 |
| -0.7 | 99.75 | 64.44 | 94.18 | 0.77 |
| -0.6 | 99.75 | 69.78 | 95.02 | 0.8 |
| -0.5 | 99.42 | 76 | 95.72 | 0.83 |
| -0.4 | 99.33 | 78.22 | 96 | 0.84 |
| -0.3 | 99.33 | 79.56 | 96.21 | 0.85 |
| -0.2 | 99.33 | 80.44 | 96.35 | 0.86 |
| -0.1 | 99.08 | 80.89 | 96.21 | 0.85 |
| 0 | 99 | 81.78 | 96.28 | 0.86 |
| 0.1 | 98.83 | 84.89 | 96.63 | 0.87 |
| 0.2 | 98.75 | 86.22 | 96.77 | 0.88 |
| **0.3** | **98.42** | **91.11** | **97.26** | **0.9** |
| 0.4 | 97.67 | 93.33 | 96.98 | 0.89 |
| 0.5 | 96.83 | 96.44 | 96.77 | 0.89 |
| 0.6 | 95.67 | 96.44 | 95.79 | 0.86 |
| 0.7 | 94.25 | 96.89 | 94.67 | 0.83 |
| 0.8 | 91.25 | 97.78 | 92.28 | 0.77 |
| 0.9 | 88.92 | 97.78 | 90.32 | 0.73 |
| 1 | 74.42 | 99.56 | 78.39 | 0.56 |

(Bold value indicates the point where overall best result was achieved)
