# Supplementary material for: Hybrid Approach for Predicting Coreceptor Used by HIV-1 from Its V3 Loop Amino Acid Sequence
Source: PLoS One. 2013 Apr 15;8(4):e61437. doi: 10.1371/journal.pone.0061437 (PMC3626595; doi:10.1371/journal.pone.0061437)
Supplement: Table S17 — The performance of Hybrid approach on Boisvert et al. [32] i.e. dskernel-R5 method dataset. The E-value “≤10−17” was used to generate the modified SVM score by Hybrid approach. (DOC) [file pone.0061437.s019.doc]

**Table S17**: The performance of Hybrid approach on Boisvert et al. [32] *i.e*. dskernel-R5 method dataset. The E-value “≤ 10^-17^” was used to generate the modified SVM score by Hybrid approach.

| **Threshold** | **Sensitivity** | **Specificity** | **Accuracy** | **MCC** |
| --- | --- | --- | --- | --- |
| -1 | 99.67 | 44.89 | 91.02 | 0.62 |
| -0.9 | 99.58 | 66.67 | 94.39 | 0.78 |
| -0.8 | 99.58 | 68.44 | 94.67 | 0.79 |
| -0.7 | 99.42 | 72.89 | 95.23 | 0.81 |
| -0.6 | 99.33 | 79.56 | 96.21 | 0.85 |
| -0.5 | 99.33 | 80.89 | 96.42 | 0.86 |
| -0.4 | 99.25 | 83.11 | 96.7 | 0.87 |
| -0.3 | 99.25 | 84.44 | 96.91 | 0.88 |
| -0.2 | 99.17 | 85.78 | 97.05 | 0.89 |
| -0.1 | 98.83 | 85.78 | 96.77 | 0.88 |
| 0 | 98.5 | 87.11 | 96.7 | 0.87 |
| 0.1 | 97.08 | 91.11 | 96.14 | 0.86 |
| 0.2 | 96.25 | 93.33 | 95.79 | 0.85 |
| **0.3** | **95.5** | **95.11** | **95.44** | **0.85** |
| 0.4 | 94.92 | 95.11 | 94.95 | 0.83 |
| 0.5 | 94.33 | 97.33 | 94.81 | 0.83 |
| 0.6 | 93.33 | 97.33 | 93.96 | 0.81 |
| 0.7 | 92.25 | 97.78 | 93.12 | 0.79 |
| 0.8 | 90.42 | 98.22 | 91.65 | 0.76 |
| 0.9 | 89 | 98.22 | 90.46 | 0.74 |
| 1 | 78.92 | 99.11 | 82.11 | 0.6 |

(Bold value indicates the point where overall best result was achieved)
