# Supplementary material for: Hybrid Approach for Predicting Coreceptor Used by HIV-1 from Its V3 Loop Amino Acid Sequence
Source: PLoS One. 2013 Apr 15;8(4):e61437. doi: 10.1371/journal.pone.0061437 (PMC3626595; doi:10.1371/journal.pone.0061437)
Supplement: Table S18 — The performance of SVM model (Learning Parameter: −z c –t 2–g 0.005 −c 7–j 1) based on Split Amino Acid Composition, on dskenel-X4 dataset. (DOC) [file pone.0061437.s020.doc]

**Table S18**: The performance of SVM model (Learning Parameter: -z c –t 2 –g 0.005 -c 7 –j 1) based on Split Amino Acid Composition, on dskenel-X4 dataset.

| **Threshold** | **Sensitivity** | **Specificity** | **Accuracy** | **MCC** |
| --- | --- | --- | --- | --- |
| -1 | 94.97 | 60.37 | 70.04 | 0.5 |
| -0.9 | 93.97 | 81.11 | 84.7 | 0.69 |
| -0.8 | 93.47 | 84.62 | 87.09 | 0.72 |
| -0.7 | 93.47 | 87.73 | 89.33 | 0.76 |
| -0.6 | 92.46 | 90.75 | 91.23 | 0.8 |
| -0.5 | 92.21 | 92.41 | 92.35 | 0.82 |
| -0.4 | 91.71 | 94.55 | 93.75 | 0.85 |
| -0.3 | 91.21 | 95.03 | 93.96 | 0.85 |
| -0.2 | 90.95 | 96.01 | 94.6 | 0.87 |
| -0.1 | 90.7 | 96.49 | 94.88 | 0.87 |
| **0** | **89.7** | **97.08** | **95.02** | **0.88** |
| 0.1 | 87.94 | 98.05 | 95.23 | 0.88 |
| 0.2 | 85.93 | 98.34 | 94.88 | 0.87 |
| 0.3 | 84.17 | 98.83 | 94.74 | 0.87 |
| 0.4 | 80.4 | 98.83 | 93.68 | 0.84 |
| 0.5 | 79.15 | 99.03 | 93.47 | 0.84 |
| 0.6 | 75.88 | 99.22 | 92.7 | 0.82 |
| 0.7 | 71.86 | 99.22 | 91.58 | 0.79 |
| 0.8 | 70.6 | 99.22 | 91.23 | 0.78 |
| 0.9 | 66.83 | 99.32 | 90.25 | 0.75 |
| 1 | 40.7 | 99.71 | 83.23 | 0.57 |

(Bold value indicates the point where overall best result was achieved)
