# Supplementary material for: Hybrid Approach for Predicting Coreceptor Used by HIV-1 from Its V3 Loop Amino Acid Sequence
Source: PLoS One. 2013 Apr 15;8(4):e61437. doi: 10.1371/journal.pone.0061437 (PMC3626595; doi:10.1371/journal.pone.0061437)
Supplement: Table S19 — The performance of Hybrid approach on Boisvert et al. [32] i.e. dskernel-X4 method dataset. The E-value “≤10−17” was used to generate the modified SVM score by Hybrid approach. (DOC) [file pone.0061437.s021.doc]

**Table S19**: The performance of Hybrid approach on Boisvert *et al.* [32] *i.e*. dskernel-X4 method dataset. The E-value “≤ 10^-17^” was used to generate the modified SVM score by Hybrid approach.

| **Threshold** | **Sensitivity** | **Specificity** | **Accuracy** | **MCC** |
| --- | --- | --- | --- | --- |
| -1 | 94.47 | 77.22 | 82.04 | 0.65 |
| -0.9 | 94.47 | 89.19 | 90.67 | 0.79 |
| -0.8 | 94.47 | 91.04 | 92 | 0.82 |
| -0.7 | 94.47 | 92.41 | 92.98 | 0.84 |
| -0.6 | 94.22 | 93.77 | 93.89 | 0.86 |
| -0.5 | 93.97 | 95.03 | 94.74 | 0.87 |
| -0.4 | 93.72 | 96.11 | 95.44 | 0.89 |
| -0.3 | 93.47 | 96.49 | 95.65 | 0.89 |
| -0.2 | 92.96 | 96.98 | 95.86 | 0.9 |
| -0.1 | 92.96 | 97.47 | 96.21 | 0.91 |
| **0** | **91.46** | **98.34** | **96.42** | **0.91** |
| 0.1 | 90.45 | 98.93 | 96.56 | 0.91 |
| 0.2 | 88.69 | 99.03 | 96.14 | 0.9 |
| 0.3 | 88.19 | 99.51 | 96.35 | 0.91 |
| 0.4 | 84.67 | 99.51 | 95.37 | 0.88 |
| 0.5 | 83.67 | 99.51 | 95.09 | 0.88 |
| 0.6 | 80.9 | 99.51 | 94.32 | 0.86 |
| 0.7 | 77.39 | 99.51 | 93.33 | 0.83 |
| 0.8 | 76.13 | 99.51 | 92.98 | 0.82 |
| 0.9 | 73.12 | 99.51 | 92.14 | 0.8 |
| 1 | 58.29 | 99.61 | 88.07 | 0.7 |

Please note that since dskernel-X4 method considered X4 as positive examples and we also considered X4 as positive data, the Hybrid approach calculated the modified SVM score by adding ‘1’ to the SAAC based SVM score if the top BLAST hit was a CXCR4; and by subtracting ‘1’ from the SAAC based SVM score if the top hit was a CCR5 sequence.
