# Supplementary material for: Hybrid Approach for Predicting Coreceptor Used by HIV-1 from Its V3 Loop Amino Acid Sequence
Source: PLoS One. 2013 Apr 15;8(4):e61437. doi: 10.1371/journal.pone.0061437 (PMC3626595; doi:10.1371/journal.pone.0061437)
Supplement: Table S20 — The performance of SVM model (Learning Parameter: −z c –t 2–g 0.01 −c 4–j 1) based on Split Amino Acid Composition, on dskenel-R5X4 dataset. (DOC) [file pone.0061437.s022.doc]

**Table S20**: The performance of SVM model (Learning Parameter: -z c –t 2 –g 0.01 -c 4 –j 1) based on Split Amino Acid Composition, on dskenel-R5X4 dataset.

| **Threshold** | **Sensitivity** | **Specificity** | **Accuracy** | **MCC** |
| --- | --- | --- | --- | --- |
| -1 | 84.97 | 51.04 | 55.16 | 0.24 |
| -0.9 | 82.66 | 76.36 | 77.12 | 0.42 |
| -0.8 | 80.35 | 82.67 | 82.39 | 0.48 |
| -0.7 | 79.19 | 88.9 | 87.72 | 0.56 |
| -0.6 | 75.14 | 93.61 | 91.37 | 0.63 |
| -0.5 | 70.52 | 97.2 | 93.96 | 0.71 |
| -0.4 | 70.52 | 97.92 | 94.6 | 0.73 |
| **-0.3** | **69.94** | **98.48** | **95.02** | **0.75** |
| -0.2 | 68.21 | 98.56 | 94.88 | 0.74 |
| -0.1 | 67.63 | 98.72 | 94.95 | 0.74 |
| 0 | 67.05 | 98.8 | 94.95 | 0.74 |
| 0.1 | 63.01 | 99.12 | 94.74 | 0.73 |
| 0.2 | 59.54 | 99.2 | 94.39 | 0.71 |
| 0.3 | 58.38 | 99.44 | 94.46 | 0.71 |
| 0.4 | 53.18 | 99.44 | 93.82 | 0.68 |
| 0.5 | 52.02 | 99.52 | 93.75 | 0.67 |
| 0.6 | 50.87 | 99.52 | 93.61 | 0.66 |
| 0.7 | 50.29 | 99.52 | 93.54 | 0.66 |
| 0.8 | 50.29 | 99.52 | 93.54 | 0.66 |
| 0.9 | 50.29 | 99.52 | 93.54 | 0.66 |
| 1 | 38.15 | 99.92 | 92.42 | 0.59 |

(Bold value indicates the point where overall best result was achieved)
