# Supplementary material for: Hybrid Approach for Predicting Coreceptor Used by HIV-1 from Its V3 Loop Amino Acid Sequence
Source: PLoS One. 2013 Apr 15;8(4):e61437. doi: 10.1371/journal.pone.0061437 (PMC3626595; doi:10.1371/journal.pone.0061437)
Supplement: Table S21 — The performance of Hybrid approach on Boisvert et al. [32] i.e. dskernel-R5X4 method dataset. The E-value “≤10−16” was used to generate the modified SVM score by Hybrid approach. (DOC) [file pone.0061437.s023.doc]

**Table S21**: The performance of Hybrid approach on Boisvert *et al.* [32] *i.e*. dskernel-R5X4 method dataset. The E-value “≤ 10^-16^” was used to generate the modified SVM score by Hybrid approach.

| **Threshold** | **Sensitivity** | **Specificity** | **Accuracy** | **MCC** |
| --- | --- | --- | --- | --- |
| -1 | 87.28 | 80.19 | 81.05 | 0.49 |
| -0.9 | 87.28 | 82.67 | 83.23 | 0.52 |
| -0.8 | 87.28 | 83.39 | 83.86 | 0.53 |
| -0.7 | 87.28 | 84.35 | 84.7 | 0.55 |
| -0.6 | 87.28 | 84.82 | 85.12 | 0.55 |
| -0.5 | 85.55 | 85.14 | 85.19 | 0.55 |
| -0.4 | 85.55 | 85.14 | 85.19 | 0.55 |
| -0.3 | 85.55 | 85.22 | 85.26 | 0.55 |
| -0.2 | 84.97 | 85.3 | 85.26 | 0.54 |
| -0.1 | 84.39 | 85.54 | 85.4 | 0.54 |
| 0 | 79.77 | 90.26 | 88.98 | 0.59 |
| 0.1 | 78.03 | 95.53 | 93.4 | 0.71 |
| 0.2 | 75.14 | 96.17 | 93.61 | 0.7 |
| 0.3 | 73.99 | 96.73 | 93.96 | 0.71 |
| 0.4 | 69.36 | 97.2 | 93.82 | 0.7 |
| 0.5 | 66.47 | 99.04 | 95.09 | 0.75 |
| **0.6** | **65.9** | **99.36** | **95.3** | **0.76** |
| 0.7 | 65.32 | 99.44 | 95.3 | 0.76 |
| 0.8 | 64.74 | 99.44 | 95.23 | 0.76 |
| 0.9 | 64.16 | 99.44 | 95.16 | 0.75 |
| 1 | 62.43 | 99.52 | 95.02 | 0.75 |

Please note that since dskernel-R5X4 method considered R5X4 as positive examples and we also included all R5X4-tropic sequences in the X4-tropic dataset, the Hybrid approach calculated the modified SVM score by adding ‘1’ to the SAAC based SVM score if the top BLAST hit was a CXCR4; and by subtracting ‘1’ from the SAAC based SVM score if the top hit was a CCR5 sequence.
