# Supplementary material for: Hybrid Approach for Predicting Coreceptor Used by HIV-1 from Its V3 Loop Amino Acid Sequence
Source: PLoS One. 2013 Apr 15;8(4):e61437. doi: 10.1371/journal.pone.0061437 (PMC3626595; doi:10.1371/journal.pone.0061437)
Supplement: Table S22 — The performance of SVM model (Learning Parameter: −z c –t 2–g 0.001 −c 7–j 1) based on Split Amino Acid Composition, on Dybowski et al. [36] dataset. This table was prepared using 10-fold cross-validation technique, as used by Dybowski et al. (DOC) [file pone.0061437.s024.doc]

**Table S22**: The performance of SVM model (Learning Parameter: -z c –t 2 –g 0.001 -c 7 –j 1) based on Split Amino Acid Composition, on Dybowski *et al*. [36] dataset. This table was prepared using 10-fold cross-validation technique, as used by Dybowski *et al.*

| **Threshold** | **Sensitivity** | **Specificity** | **Accuracy** | **MCC** |
| --- | --- | --- | --- | --- |
| -1 | 94.58 | 89.31 | 89.98 | 0.68 |
| -0.9 | 94.58 | 92.09 | 92.41 | 0.74 |
| -0.8 | 93.98 | 94.09 | 94.08 | 0.78 |
| -0.7 | 92.77 | 95.57 | 95.22 | 0.81 |
| -0.6 | 90.96 | 96.7 | 95.98 | 0.83 |
| -0.5 | 90.96 | 97.39 | 96.58 | 0.85 |
| -0.4 | 90.96 | 97.83 | 96.96 | 0.87 |
| -0.3 | 89.16 | 98.26 | 97.11 | 0.87 |
| **-0.2** | **89.16** | **98.7** | **97.49** | **0.89** |
| -0.1 | 88.55 | 98.87 | 97.57 | 0.89 |
| 0 | 86.14 | 98.96 | 97.34 | 0.88 |
| 0.1 | 82.53 | 99.13 | 97.04 | 0.86 |
| 0.2 | 80.72 | 99.13 | 96.81 | 0.85 |
| 0.3 | 75.9 | 99.22 | 96.28 | 0.82 |
| 0.4 | 74.7 | 99.22 | 96.13 | 0.81 |
| 0.5 | 72.89 | 99.3 | 95.98 | 0.81 |
| 0.6 | 68.67 | 99.3 | 95.44 | 0.78 |
| 0.7 | 64.46 | 99.57 | 95.14 | 0.76 |
| 0.8 | 60.84 | 99.57 | 94.68 | 0.74 |
| 0.9 | 56.02 | 99.74 | 94.23 | 0.71 |
| 1 | 49.4 | 99.74 | 93.39 | 0.66 |

(Bold value indicates the point where overall best result was achieved)
