# Supplementary material for: Hybrid Approach for Predicting Coreceptor Used by HIV-1 from Its V3 Loop Amino Acid Sequence
Source: PLoS One. 2013 Apr 15;8(4):e61437. doi: 10.1371/journal.pone.0061437 (PMC3626595; doi:10.1371/journal.pone.0061437)
Supplement: Table S23 — The performance of Hybrid approach on Dybowski et al. [36] dataset. The E-value “≤10−15” was used to generate the modified SVM score by Hybrid approach. (DOC) [file pone.0061437.s025.doc]

**Table S23**: The performance of Hybrid approach on Dybowski *et al*. [36] dataset. The E-value “≤ 10^-15^” was used to generate the modified SVM score by Hybrid approach.

| **Threshold** | **Sensitivity** | **Specificity** | **Accuracy** | **MCC** |
| --- | --- | --- | --- | --- |
| 1 | 85.54 | 99.74 | 97.95 | 0.9 |
| 0.9 | 88.55 | 99.74 | 98.33 | 0.92 |
| 0.8 | 89.16 | 99.65 | 98.33 | 0.92 |
| 0.7 | 89.16 | 99.65 | 98.33 | 0.92 |
| 0.6 | 90.96 | 99.65 | 98.56 | 0.93 |
| 0.5 | 90.96 | 99.65 | 98.56 | 0.93 |
| 0.4 | 90.96 | 99.65 | 98.56 | 0.93 |
| 0.3 | 92.77 | 99.65 | 98.79 | 0.94 |
| 0.2 | 93.98 | 99.65 | 98.94 | 0.95 |
| **0.1** | **94.58** | **99.65** | **99.01** | **0.95** |
| 0 | 94.58 | 99.57 | 98.94 | 0.95 |
| -0.1 | 94.58 | 99.48 | 98.86 | 0.95 |
| -0.2 | 94.58 | 99.22 | 98.63 | 0.94 |
| -0.3 | 94.58 | 99.04 | 98.48 | 0.93 |
| -0.4 | 95.18 | 98.78 | 98.33 | 0.93 |
| -0.5 | 95.78 | 98.78 | 98.41 | 0.93 |
| -0.6 | 95.78 | 98.52 | 98.18 | 0.92 |
| -0.7 | 95.78 | 98.52 | 98.18 | 0.92 |
| -0.8 | 96.39 | 98.52 | 98.25 | 0.92 |
| -0.9 | 96.99 | 98.52 | 98.33 | 0.93 |
| -1 | 96.99 | 98.18 | 98.03 | 0.92 |

Please note that since Dybowski et al [36] method considered X4-sequences as positive examples, the hybrid approach also calculated modified SVM score by adding ‘1’ to the SAAC based SVM score if the top BLAST hit was CXCR4; and by subtracting ‘1’ from the SAAC based SVM score if the top BLAST hit was a CCR5 sequence.
