# Supplementary material for: Hybrid Approach for Predicting Coreceptor Used by HIV-1 from Its V3 Loop Amino Acid Sequence
Source: PLoS One. 2013 Apr 15;8(4):e61437. doi: 10.1371/journal.pone.0061437 (PMC3626595; doi:10.1371/journal.pone.0061437)
Supplement: Table S24 — The performance of SVM model (Learning Parameter: −z c –t 2–g 0.005 −c 8–j 1) based on Split Amino Acid Composition, on Xu et al. [30] dataset. (DOC) [file pone.0061437.s026.doc]

**Table S24**: The performance of SVM model (Learning Parameter: -z c –t 2 –g 0.005 -c 8 –j 1) based on Split Amino Acid Composition (SAAC), on Xu *et al*. [30] dataset.

| **Threshold** | **Sensitivity** | **Specificity** | **Accuracy** | **MCC** |
| --- | --- | --- | --- | --- |
| -1 | 100 | 45.57 | 86.6 | 0.62 |
| -0.9 | 100 | 64.56 | 91.28 | 0.76 |
| -0.8 | 99.79 | 67.72 | 91.9 | 0.78 |
| -0.7 | 99.38 | 71.52 | 92.52 | 0.79 |
| -0.6 | 99.38 | 74.05 | 93.15 | 0.81 |
| -0.5 | 99.38 | 75.32 | 93.46 | 0.82 |
| -0.4 | 99.38 | 77.22 | 93.93 | 0.83 |
| -0.3 | 98.97 | 78.48 | 93.93 | 0.83 |
| -0.2 | 98.97 | 84.18 | 95.33 | 0.87 |
| **-0.1** | **98.76** | **87.34** | **95.95** | **0.89** |
| 0 | 97.93 | 87.34 | 95.33 | 0.87 |
| 0.1 | 97.31 | 87.34 | 94.86 | 0.86 |
| 0.2 | 96.07 | 87.97 | 94.08 | 0.84 |
| 0.3 | 94.83 | 88.61 | 93.3 | 0.82 |
| 0.4 | 93.6 | 88.61 | 92.37 | 0.8 |
| 0.5 | 91.74 | 89.24 | 91.12 | 0.78 |
| 0.6 | 89.26 | 89.24 | 89.25 | 0.74 |
| 0.7 | 86.16 | 90.51 | 87.23 | 0.7 |
| 0.8 | 82.02 | 92.41 | 84.58 | 0.67 |
| 0.9 | 77.69 | 93.04 | 81.46 | 0.62 |
| 1 | 67.36 | 93.67 | 73.83 | 0.53 |

(Bold value indicates the point where overall best result was achieved)
