# Supplementary material for: Hybrid Approach for Predicting Coreceptor Used by HIV-1 from Its V3 Loop Amino Acid Sequence
Source: PLoS One. 2013 Apr 15;8(4):e61437. doi: 10.1371/journal.pone.0061437 (PMC3626595; doi:10.1371/journal.pone.0061437)
Supplement: Table S25 — The performance of Hybrid approach on Xu et al. [30] dataset. The E-value “≤10−17” was used to generate the modified SVM score by Hybrid approach. (DOC) [file pone.0061437.s027.doc]

**Table S25**: The performance of Hybrid approach on Xu *et al*. [30] dataset. The E-value “≤ 10^-17^” was used to generate the modified SVM score by Hybrid approach.

| **Threshold** | **Sensitivity** | **Specificity** | **Accuracy** | **MCC** |
| --- | --- | --- | --- | --- |
| -1 | 100 | 57.59 | 89.56 | 0.71 |
| -0.9 | 100 | 72.15 | 93.15 | 0.81 |
| -0.8 | 99.79 | 75.32 | 93.77 | 0.83 |
| -0.7 | 99.59 | 79.11 | 94.55 | 0.85 |
| -0.6 | 99.59 | 80.38 | 94.86 | 0.86 |
| -0.5 | 99.59 | 81.01 | 95.02 | 0.86 |
| -0.4 | 99.59 | 82.28 | 95.33 | 0.87 |
| -0.3 | 99.38 | 83.54 | 95.48 | 0.88 |
| -0.2 | 99.38 | 87.34 | 96.42 | 0.9 |
| **-0.1** | **99.17** | **90.51** | **97.04** | **0.92** |
| 0 | 98.76 | 90.51 | 96.73 | 0.91 |
| 0.1 | 98.14 | 90.51 | 96.26 | 0.9 |
| 0.2 | 97.52 | 91.14 | 95.95 | 0.89 |
| 0.3 | 96.28 | 91.77 | 95.17 | 0.87 |
| 0.4 | 95.25 | 92.41 | 94.55 | 0.86 |
| 0.5 | 94.42 | 93.04 | 94.08 | 0.85 |
| 0.6 | 92.77 | 93.04 | 92.83 | 0.82 |
| 0.7 | 90.91 | 93.67 | 91.59 | 0.8 |
| 0.8 | 87.81 | 94.3 | 89.41 | 0.76 |
| 0.9 | 85.33 | 94.3 | 87.54 | 0.72 |
| 1 | 80.17 | 94.94 | 83.8 | 0.67 |

(Bold value indicates the point where overall best result was achieved)
