# Supplementary material for: Hybrid Approach for Predicting Coreceptor Used by HIV-1 from Its V3 Loop Amino Acid Sequence
Source: PLoS One. 2013 Apr 15;8(4):e61437. doi: 10.1371/journal.pone.0061437 (PMC3626595; doi:10.1371/journal.pone.0061437)
Supplement: Table S26 — The TXT (raw values) showing residue frequencies of R5- and X4-tropic sequences generated by using two-sample logo method. (DOC) [file pone.0061437.s028.doc]

**Table S26**: The TXT (raw values) showing residue frequencies of R5- and X4-tropic sequences generated by using two-sample logo based approach.

| **Position** | **Symbol** | **Frequency** | **P-value** |
| --- | --- | --- | --- |
| 2 | I | 0.0399453551912568 | 0.028681 |
| 2 | M | -0.033625843780135 | 3.0e-06 |
| 4 | L | 0.0111475409836066 | 0.032138 |
| 4 | P | -0.0270572163291546 | 0.001664 |
| 5 | F | -0.0520941819350691 | 0.0 |
| 5 | G | -0.0410768241722919 | 0.028834 |
| 5 | H | 0.01525393764063 | 0.022607 |
| 5 | N | 0.119177113468338 | 1.5e-05 |
| 5 | Y | -0.0395274831243973 | 0.0 |
| 6 | I | -0.00490196078431373 | 0.005837 |
| 6 | K | -0.0526100932176149 | 0.0 |
| 6 | N | 0.115131790421086 | 0.0 |
| 6 | R | -0.00735294117647059 | 0.000728 |
| 6 | Y | -0.0245098039215686 | 0.0 |
| 7 | A | -0.00735294117647059 | 0.000765 |
| 7 | H | -0.0133944069431051 | 0.000168 |
| 7 | I | -0.00669720347155256 | 0.00792 |
| 7 | K | -0.0336579877852781 | 0.0 |
| 7 | N | 0.157486338797814 | 0.0 |
| 7 | P | -0.0147058823529412 | 2.0e-06 |
| 7 | S | -0.00963195114111218 | 0.010705 |
| 7 | T | -0.0220588235294118 | 0.0 |
| 7 | Y | -0.0396994535519126 | 0.0 |
| 8 | I | -0.0756364513018322 | 0.0 |
| 8 | K | -0.0390437158469945 | 0.0 |
| 8 | M | -0.00735294117647059 | 0.000803 |
| 8 | Q | -0.00735294117647059 | 0.000803 |
| 8 | R | -0.00718097074895532 | 0.045041 |
| 8 | T | 0.152068466730955 | 0.0 |
| 8 | V | -0.0151896496303439 | 0.000145 |
| 9 | E | -0.00980392156862745 | 9.8e-05 |
| 9 | I | -0.016468981035037 | 0.010835 |
| 9 | K | -0.044429443908711 | 0.0 |
| 9 | Q | -0.00490196078431373 | 0.005947 |
| 9 | R | 0.0884185149469624 | 0.0 |
| 9 | S | 0.0119752491160399 | 0.049209 |
| 10 | A | -0.00735294117647059 | 0.000806 |
| 10 | I | -0.00980392156862745 | 0.000108 |
| 10 | K | 0.0956557377049181 | 0.000123 |
| 10 | N | -0.00669720347155256 | 0.008244 |
| 10 | T | -0.045591449694632 | 0.008854 |
| 11 | G | -0.062727418836387 | 0.002085 |
| 11 | H | -0.00490196078431373 | 0.006017 |
| 11 | K | -0.0122549019607843 | 1.3e-05 |
| 11 | Q | -0.0115991642558663 | 0.000169 |
| 11 | R | -0.278412086145934 | 0.0 |
| 11 | S | 0.392062037929926 | 0.0 |
| 12 | F | -0.0140501446480231 | 2.4e-05 |
| 12 | I | 0.209561234329797 | 0.0 |
| 12 | L | -0.0396994535519126 | 0.0 |
| 12 | R | -0.00980392156862745 | 9.8e-05 |
| 12 | S | -0.00490196078431373 | 0.005958 |
| 12 | T | -0.0117389906782385 | 0.035249 |
| 12 | V | -0.128871745419479 | 0.0 |
| 13 | A | -0.00718097074895532 | 0.042959 |
| 13 | F | -0.00735294117647059 | 0.000749 |
| 13 | G | -0.0334538733526197 | 1.6e-05 |
| 13 | H | 0.151623272259724 | 0.0 |
| 13 | I | -0.00735294117647059 | 0.000749 |
| 13 | N | 0.0718193506910961 | 0.0 |
| 13 | P | 0.0905560912889746 | 0.0 |
| 13 | S | -0.064080681452909 | 0.0 |
| 13 | T | -0.103232079717133 | 0.0 |
| 13 | Y | -0.0615863066538091 | 0.0 |
| 14 | A | -0.00490196078431373 | 0.006186 |
| 14 | H | -0.0122549019607843 | 1.4e-05 |
| 14 | I | 0.0853567984570878 | 0.000402 |
| 14 | L | -0.0407537769206043 | 0.009629 |
| 14 | P | -0.0147058823529412 | 2.0e-06 |
| 14 | T | -0.01650112504018 | 4.0e-06 |
| 14 | V | -0.0128784956605593 | 0.032896 |
| 15 | A | 0.0303359048537448 | 0.000677 |
| 15 | I | -0.0318627450980392 | 0.0 |
| 15 | S | -0.00490196078431373 | 0.006211 |
| 16 | A | -0.00735294117647059 | 0.000788 |
| 16 | G | -0.0318627450980392 | 0.0 |
| 16 | P | 0.0826133076181292 | 0.0 |
| 16 | Q | -0.0297235615557699 | 0.0 |
| 16 | T | -0.00490196078431373 | 0.006165 |
| 17 | G | 0.0426342012214721 | 0.0 |
| 17 | L | -0.00490196078431373 | 0.006211 |
| 17 | P | -0.0147058823529412 | 2.0e-06 |
| 17 | Q | -0.00980392156862745 | 0.000106 |
| 17 | R | -0.00783670845387335 | 0.019242 |
| 17 | S | -0.00490196078431373 | 0.006211 |
| 18 | H | -0.0140501446480231 | 2.3e-05 |
| 18 | K | 0.0415477338476374 | 0.001196 |
| 18 | Q | 0.0777499196399871 | 0.006122 |
| 18 | R | -0.0887672774027644 | 0.000662 |
| 19 | A | 0.254561234329797 | 0.0 |
| 19 | K | -0.0140501446480231 | 2.6e-05 |
| 19 | Q | -0.0147058823529412 | 2.0e-06 |
| 19 | R | -0.0182963677274188 | 4.0e-06 |
| 19 | V | -0.200723240115718 | 0.0 |
| 19 | Y | -0.0122549019607843 | 1.4e-05 |
| 20 | A | -0.0245098039215686 | 0.0 |
| 20 | F | 0.193252973320476 | 0.0 |
| 20 | V | -0.0864079074252652 | 0.0 |
| 20 | W | -0.0344969463195114 | 0.004653 |
| 20 | Y | -0.0298633879781421 | 5.4e-05 |
| 21 | H | -0.0341096110575378 | 9.0e-06 |
| 21 | I | -0.00490196078431373 | 0.006026 |
| 21 | L | -0.0147058823529412 | 2.0e-06 |
| 21 | S | -0.0122549019607843 | 1.3e-05 |
| 21 | Y | 0.0962664738026358 | 0.0 |
| 22 | A | 0.240167148826744 | 0.0 |
| 22 | H | -0.0122549019607843 | 1.4e-05 |
| 22 | K | -0.054545162327226 | 0.0 |
| 22 | T | -0.114284795885567 | 2.0e-06 |
| 22 | V | -0.0101157184185149 | 0.025614 |
| 22 | Y | -0.0189521054323369 | 1.0e-06 |
| 23 | A | -0.0380118932819029 | 2.0e-05 |
| 23 | H | -0.00669720347155256 | 0.008162 |
| 23 | M | -0.0145339119254259 | 0.00052 |
| 23 | R | -0.0130504660880746 | 0.014734 |
| 23 | S | -0.00718097074895532 | 0.044738 |
| 23 | T | 0.0803776920604307 | 0.0 |
| 24 | D | -0.0323143683702989 | 1.6e-05 |
| 24 | E | -0.0705303760848602 | 0.0 |
| 24 | G | 0.236488267438123 | 0.0 |
| 24 | K | -0.0375602700096432 | 0.0 |
| 24 | N | 0.0164255866280939 | 0.049538 |
| 24 | R | -0.0573400835744134 | 0.0 |
| 24 | S | -0.00490196078431373 | 0.006072 |
| 24 | T | -0.0427740276438444 | 0.0 |
| 25 | D | 0.214337833494053 | 0.0 |
| 25 | E | 0.104905175184828 | 4.3e-05 |
| 25 | K | -0.101425586628094 | 0.0 |
| 25 | N | -0.0187479909996786 | 0.00835 |
| 25 | Q | -0.0945676631308261 | 0.0 |
| 25 | R | -0.0604146576663452 | 0.0 |
| 26 | G | -0.00490196078431373 | 0.006055 |
| 26 | K | -0.00783670845387335 | 0.018698 |
| 27 | E | -0.00735294117647059 | 0.000775 |
| 27 | I | 0.173558341369335 | 0.0 |
| 27 | K | -0.00914818386370942 | 0.001188 |
| 27 | T | -0.0635969141755063 | 0.0 |
| 27 | V | -0.0704660880745741 | 0.0 |
| 28 | K | -0.00490196078431373 | 0.006148 |
| 29 | G | -0.0115991642558663 | 0.000173 |
| 29 | N | 0.0676792028286725 | 0.002426 |
| 29 | R | -0.00914818386370942 | 0.001186 |
| 29 | S | -0.00669720347155256 | 0.007998 |
| 29 | T | -0.00735294117647059 | 0.000774 |
| 29 | Y | -0.017156862745098 | 0.0 |
| 30 | I | 0.0329379620700739 | 0.000635 |
| 30 | N | -0.00490196078431373 | 0.006105 |
| 30 | T | -0.00897621343619415 | 0.024269 |
| 30 | V | -0.00963195114111218 | 0.010865 |
| 31 | G | -0.0133944069431051 | 0.00017 |
| 31 | K | -0.0172966891674703 | 0.002615 |
| 31 | R | 0.0388396014143363 | 4.0e-06 |
| 32 | E | 0.020983606557377 | 0.003254 |
| 32 | K | -0.131666666666667 | 0.0 |
| 32 | Q | 0.257205078752813 | 0.0 |
| 32 | R | -0.150692703310833 | 0.0 |
| 33 | A | 0.0168129218900676 | 0.001225 |
| 33 | P | -0.00980392156862745 | 0.000106 |
| 34 | H | 0.0682754741240759 | 0.005148 |
| 34 | S | 0.0111475409836066 | 0.032145 |
| 34 | Y | -0.100159112825458 | 1.5e-05 |
